# Age among women and men, time to pregnancy and risk of miscarriage

**DOI:** 10.1186/s12916-025-04462-8

**Published:** 2025-11-17

**Authors:** Aline J. Boxem, Sophia M. Blaauwendraad, Annemarie G. M. G. J. Mulders, Eline L. Bekkers, Romy Gaillard, Vincent W. V. Jaddoe

**Affiliations:** 1 The Generation R Study Group, Erasmus MC, University Medical Center, Rotterdam, the Netherlands; 2 Department of Pediatrics, Erasmus MC, University Medical Center, Rotterdam, the Netherlands; 3 Department of Obstetrics and Gynaecology, Erasmus MC, University Medical Center, Rotterdam, the Netherlands

**Keywords:** Reproductive age, Couples, Preconception, Fertility, Fecundability, Infertility, Miscarriage

## Abstract

**Background:**

Older age of women is strongly associated with reduced fertility and early pregnancy complications. These associations might also be present across the full range of reproductive age of both women and men. We assessed the associations of age of women and men with time to pregnancy and miscarriage risk.

**Methods:**

This population-based prospective cohort study was conducted in Rotterdam, the Netherlands. A total of 3604 women and their partners were included from the preconception period onwards with follow-up until birth. For this study, participants were followed until conception or 22 weeks of pregnancy for the time to pregnancy and miscarriage analyses, respectively. Outcomes included fecundability, defined as the per-month probability of conceiving; infertility, defined as a time to pregnancy or duration of pursuing pregnancy of more than 12 months or use of assisted reproductive technology; and miscarriage, defined as pregnancy loss before 22 weeks of gestation.

**Results:**

In total, 18.1% of the population was infertile and 12.7% of all recognized pregnancies led to a miscarriage. As compared to women aged 30.0–34.9 years, those aged < 25.0, 25.0–29.9, and ≥ 40.0 years had increased odds of infertility (odds ratio (OR) 2.14 (95% confidence interval (CI), 1.60–2.87); OR 1.27 (95% CI, 1.05–1.53); and OR 2.00 (95% CI, 1.05–3.80), respectively). Women aged 35.0–39.9 years had no increased odds of infertility. Similar results were observed for fecundability. As compared to women aged 30.0–34.9 years at conception, those aged 35.0–39.9 and ≥ 40.0 years had strongly increased odds of miscarriage (OR 2.03 (95% CI, 1.51–2.72) and 4.24 (95% CI, 2.45–7.36), respectively). As compared to men aged 30.0–34.9 years, those aged < 25.0 years had lower fecundability (fecundability ratio (FR) 0.71 (95% CI, 0.58–0.87)). As compared to men aged 30.0–34.9 years, those aged ≥ 40.0 years had the highest odds of miscarriage (OR, 2.09 (95% CI, 1.39–3.14)).

**Conclusions:**

Age among both women and their partners is a strong risk factor for adverse fertility and early pregnancy outcomes. Strategies optimizing age at which couples start family planning, focusing on both women and men, will have a great individual and public health impact.

**Supplementary Information:**

The online version contains supplementary material available at 10.1186/s12916-025-04462-8.

## Background

Age at which couples in western countries start contemplating a pregnancy seems to be slowly increasing [1, 2]. This may be due to different and complex interactions between cultural and social factors, such as career choices and societal expectations, and may have important consequences for fertility and pregnancy outcomes [3–6]. Older age among women is a well-known and strong risk factor for adverse reproductive outcomes, such as lower oocyte quality, contributing to impaired fertility and miscarriage risk [7–16]. Older age among women is also linked to increased risks of pregnancy complications, such as gestational hypertensive disorders and diabetes, preterm birth, and congenital anomalies [16–23]. Furthermore, results of a small number of studies suggest that older age among men is associated with lower sperm quality, which may contribute to adverse reproductive outcomes, congenital anomalies, and perinatal mortality [12, 13, 24–28]. Although much is known about the effects of older age among women on infertility and miscarriage risks, less is known about the potential effects of younger age among women and about the combined effects of age among both women and men on these outcomes. Better knowledge about the age effects across the full reproductive age range among both women and men separately and combined in the general population on fertility and early pregnancy outcomes is urgently needed for optimization of reproductive counseling and effective and balanced preventive strategies for couples who start family planning.

We hypothesized that older reproductive age is related to a prolonged time to pregnancy and increased miscarriage risk. We assessed, in a population-based prospective cohort study from the preconception or early pregnancy periods onwards among women and their partners, the separate and combined associations of the age of women and men with fecundability, risk of infertility (defined as a time to pregnancy of > 12 months or fertility treatment), and risk of miscarriage.

## Methods

### Study design

**Fig. 1:**
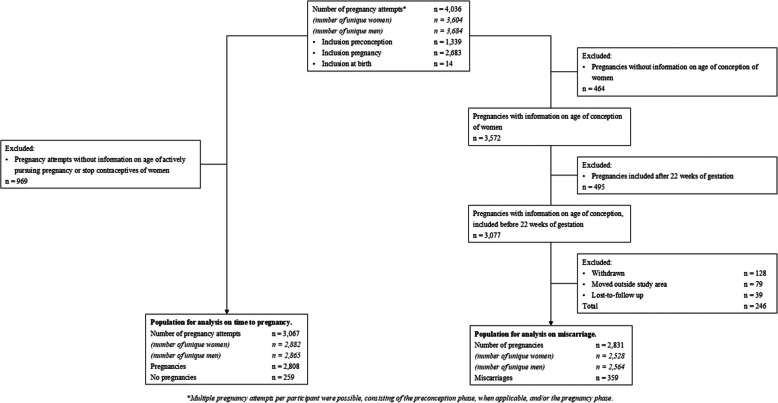
Flowchart of participants included in the analyses

This study was embedded in the Generation R *Next* Study, a population-based prospective cohort study from the preconception period onwards in Rotterdam, the Netherlands [29]. Women and their partners in the general population were eligible if they were ≥ 18 years old, living in Rotterdam and were actively trying to conceive or pregnant. Women and their partners were recruited through various approaches: an information folder sent out to all inhabitants living in the city of Rotterdam, information about the study through their general practitioner, pharmacy, or obstetric caregiver, or acquaintances, and social media, posters and news outlets. Couples were included in preconception or pregnancy between August 2017 and July 2021. Inclusion was aimed for in preconception or early pregnancy, but was allowed until delivery. If pregnant at the end of the study inclusion period, couples were followed up until pregnancy ended. This follow-up was independent of an obstetric caregiver, but performed by the study group. If women were not pregnant at the end of the pregnancy enrollment period, their end date of actively pursuing pregnancy was the study end date (Additional file 1: Fig. S1). In total, 33.2% of the inclusions were in preconception, 52.8% in the first trimester, and 14% after the first trimester. Study approval was obtained by the Medical Ethical Committee of the Erasmus University Medical Center, Rotterdam (MEC 2016–589, NL57828.078.16). Written informed consent was obtained from participating women and men. Couples could participate more than once for different preconception or pregnancy episodes. In total, there were 4036 participant episodes from 3604 unique women, leading to 3577 pregnancies of 3200 unique women at the end of the study. In total, 71.3% (*n* = 2568) of the partners participated in the study. For the time to pregnancy analyses, we excluded episodes without a known age at pursuing pregnancy (*n* = 969). Fecundability and infertility analyses were based on 3067 episodes (2882 women). For the miscarriage analyses, episodes without a known age at conception were excluded (*n* = 464). Miscarriage analyses were based on 2831 episodes (2528 women) (Fig. 1).

### Age among women and men

Information on age was obtained through questionnaires at enrollment. We used self-reported questions regarding the date at which women and men started trying to conceive or, if missing, the date at which women refrained from using contraceptives (start date pursuing pregnancy). The first day of the last menstrual period was obtained from the obstetric caregiver, based on the due date calculated at ultrasonography. Age at pursuing pregnancy of women and men was calculated by subtracting the birth date of women and men from the start date of actively trying to conceive. Age at conception was calculated by subtracting the birth date from the first day of the last menstrual period. Age was categorized in < 25.0 years, 25.0–29.9 years, 30.0–34.9 years, 35.0–39.9 years and ≥ 40.0 years. Age of women and men combined was categorized in both aged < 35 years, only men aged ≥ 35 years, only women aged ≥ 35 years, and both aged ≥ 35 years.

### Time to pregnancy and miscarriage

As described earlier, time to pregnancy and mode of conception were assessed through questionnaires in preconception and early pregnancy [29]. We used questions regarding the date at which women started trying to conceive and refrained from using contraceptives (start date pursuing pregnancy) or the date of assisted reproductive technology, including intrauterine insemination (IUI), ovulation induction, in vitro fertilization (IVF), and intracytoplasmic sperm injection. Time to pregnancy was calculated as the difference between the start date of pursuing pregnancy and the first day of the last menstrual period. When participants were included during the preconception period, they informed the study group when they obtained a self-performed positive pregnancy test and were asked what their first day of the last menstrual period was. Also, all women without information about a conception received a questionnaire about fertility outcomes 1 year after their enrollment. If participants were included during the first trimester, the first day of the last menstrual period was obtained during their registration visit and in questionnaires. The first day of the last menstrual period was then reconfirmed during the ultrasounds at the 7, 9, or 11 weeks visits to the research center. In women who did not conceive, the duration of pursuing pregnancy was calculated from the start date of pursuing pregnancy and the last day of study participation (i.e., the date when voluntarily withdrawing) or the study end date. Inclusion of participants ended on December 31, 2020, and non-pregnant women were followed up until July 1, 2021 (study end date). Ongoing pregnancies at that point were followed up until birth. Time to pregnancy was assessed both continuously and categorized. Continuous time to pregnancy was used to assess fecundability. Fecundability was defined as the per-month probability of conceiving, defined as 4 weeks (28 days), to represent a regular menstrual cycle length. Time to pregnancy was categorized as fertile (time to pregnancy of 0–52 weeks, corresponding to 0–12 months) or infertile (time to pregnancy or, in case of not conceiving, duration of pursuing pregnancy of > 52 weeks, corresponding to > 12 months, or the use of assisted reproductive technology), according to the clinical definition [30–33]. Spontaneous miscarriage was defined as a pregnancy loss before 22 weeks of gestation [34]. Date of miscarriage was obtained from the obstetric caregiver. Gestational age at miscarriage was based on the first day of the last menstrual period, the due date based on ultrasonography, date of IUI, or embryo implant day minus 14 days in case of IVF or intracytoplasmic sperm injection.

### Covariates

Information on ethnicity and educational level was obtained through questionnaires at enrollment. Ethnicity was assessed by the country of birth of the participant and her or his parents. If one of the parents of the participant was born abroad, participants were classified as of non-Dutch ethnic origin [35, 36]. If both parents were born abroad, the country of birth of the mother defined the ethnicity of the participant. Information on body mass index (BMI) was obtained from height and weight measured during the preconceptional visit or obtained via questionnaires, and if prepregnancy weight was missing, supplemented with the weight measurements at the early pregnancy visits. Height was measured at the first visit to our research center. Information on smoking and alcohol consumption in women and men was assessed through questionnaires in preconception and/or early pregnancy. Additionally, information on parity and history of previous miscarriage was assessed through questionnaires completed during preconception and/or early pregnancy.

### Statistical analysis

First, we performed a non-response analysis comparing characteristics of women with and without information on time to pregnancy or miscarriage by using *t*-tests, Mann–Whitney *U*-tests, chi-square tests, or Fisher’s exact tests. Second, we examined the associations of age at pursuing pregnancy among women and men with fecundability using Cox proportional hazards models (R package *survival*). We first excluded episodes without information on time to pregnancy or duration of pursuing pregnancy from the total study population. We then constructed the model. The outcome was conception or no conception. Time-to-event for fecundability was measured in months from the start of pursuing pregnancy to conception for women who conceived. For women who did not conceive during follow-up, time-to-event was measured to the date of last reported pregnancy attempt, defined as the date of participant’s voluntary study withdrawal or formal study end date, whichever occurred first. These observations were right-censored. Women who underwent assisted conception were excluded from the fecundability analysis due to unknown or unreliable time to pregnancy. The resulting hazard ratio (HR) from the Cox model represents the fecundability ratio (FR) (Additional file 1: Supplemental Methods). Third, we examined the associations of age at conception among women and men with time to miscarriage using Cox proportional hazards models (R package *survival*). We first excluded participants who were included after 22 weeks of pregnancy, those without a known pregnancy outcome and/or with a known pregnancy outcome but missing information on gestational age at pregnancy outcome from the total study population. We then constructed the model. The outcome was miscarriage or no miscarriage. Time-to-event for miscarriage was based on gestational age in weeks. Pregnancies ending in live birth or stillbirth were censored at 22 weeks. Ectopic pregnancies and terminations were seen as “no miscarriage” due to the different pathophysiological mechanisms involved and were treated as censoring events at the gestational age at which the pregnancy ended. For both models, we checked the proportional hazard assumptions of the covariates using plots of Schoenfeld residuals, assessed linearity of all associations using Martingale residuals, and assessed influentials using deviance residuals. Fourth, we examined the associations of age at pursuing pregnancy among women and men with the odds of infertility and age at conception among women and men with the odds of miscarriage using logistic regression models. All models were first analyzed in univariate models and second in multivariate models with adjustment for potential confounders. We performed all models on multiple imputed datasets using Rubin’s rules to pool estimates. Cluster-robust standard errors were calculated using a sandwich estimator with clustering at the individual level to account for within-group correlation for participants with multiple episodes. Hazards ratios, odds ratios, and their corresponding 95% confidence intervals were derived from the pooled effect estimates and standard errors. Potential confounders were selected a priori based on a directed acyclic graph (DAG) and associations of the exposure and outcome in existing literature (Additional file 1: Fig. S2–S3) and included ethnicity, education level, BMI, smoking, alcohol consumption, parity, and history of miscarriage. We performed five sensitivity analyses. First, since some women participated more than once in the study, we repeated the analyses including only their first episode in the study. Second, to assess whether any association was driven or influenced by assisted reproductive technology, we excluded participants who underwent assisted conception. Third, since the time to pregnancy distribution was right-skewed, we repeated the Cox analysis excluding the top 5% of the time to pregnancy distribution to make sure that the right-skewed distribution would not influence our results. Fourth, to account for potential confounding by partners’ age, we repeated the analyses adjusting for age of partner. Fifth, to account for the possible difference between the preconceptionally and prenatally included cohorts, we repeated the analyses including only the preconceptionally included cohort. Missing values of covariates were imputed using multiple imputation by chained equations to reduce potential bias due to missing information of covariates (R package *mice*). The percentage of missing values for covariates ranged from 0% to 21.2% for history of miscarriage in women. Analyses were performed using R Statistical Software, version 4.4.1.

## Results

### Population characteristics

Table 1 and Tables S3–S7 from Additional file 1 show the overall characteristics and characteristics stratified per age group and inclusion moment for the time to pregnancy and miscarriage populations. The median time to pregnancy was 3.8 months (95% range, 0.0–67.9 months). In total, 18.1% (*n* = 554) of the 3067 episodes were infertile. In total, 12.7% (*n* = 359) of the 2831 recognized pregnancies led to a miscarriage.

**Table 1:** Characteristics of Episodes of Included Participants^a^

Characteristic	Episodes, No. (%)			
	**Time to pregnancy**		**Miscarriage**	
	**Women**	**Men**	**Women**	**Men**
	***N* = 3067**^**b**^	***N* = 2464**	***N* = 2831**^**c**^	***N* = 2392**
Age at pursuing pregnancy, median (IQR), years	30.8 (28.2, 33.3)	32.1 (29.4, 35.4)	30.9 (28.5, 33.3)	32.2 (29.4, 35.4)
< 25.0	277 (9.0)	134 (5.4)	187 (8.0)	107 (5.2)
25.0–29.9	1003 (32.7)	605 (24.6)	758 (32.5)	510 (24.7)
30.0–34.9	1339 (43.7)	1035 (42.0)	1062 (45.6)	861 (41.8)
35.0–39.9	404 (13.2)	479 (19.4)	297 (12.8)	408 (19.8)
≥ 40.0	44 (1.4)	211 (8.6)	25 (1.1)	175 (8.5)
*Missing*	*0 (0.0)*	*0 (0.0)*	*502 (17.7)*	*331 (13.8)*
Age at conception, median (IQR), years	31.6 (29.2, 34.3)	33.2 (30.3, 36.6)	31.5 (28.9, 34.3)	33.3 (30.2, 36.5)
<25.0	150 (5.3)	77 (3.2)	186 (6.6)	98 (4.1)
25.0–29.9	777 (27.7)	484 (20.0)	771 (27.2)	483 (20.2)
30.0–34.9	1294 (46.1)	995 (41.0)	1289 (45.5)	947 (39.6)
35.0–39.9	514 (18.3)	576 (23.8)	508 (17.9)	579 (24.2)
≥ 40.0	73 (2.6)	292 (12.0)	77 (2.7)	285 (11.9)
*Missing*	*259 (8.4)*	*40 (1.6)*	*0 (0.0)*	*0 (0.0)*
Ethnicity				
Dutch	1887 (61.9)	1586 (65.3)	1647 (63.1)	1455 (65.2)
European^c^	313 (10.3)	202 (8.3)	256 (9.8)	180 (8.1)
Non-European^d^	849 (27.8)	641 (26.4)	708 (27.1)	598 (26.8)
* Missing*	*18 (0.6)*	*35 (1.4)*	*220 (7.8)*	*159 (6.6)*
Education level				
No, primary or secondary education finished	866 (28.6)	857 (35.0)	736 (28.4)	783 (34.9)
Higher education finished	2165 (71.4)	1589 (65.0)	1856 (71.6)	1459 (65.1)
* Missing*	*36 (1.2)*	*18 (0.7)*	*239 (8.4)*	*150 (6.3)*
BMI, median (IQR), kg/m^2^	23.5 (21.2, 26.5)	24.9 (23.0, 27.4)	23.5 (21.3, 26.7)	25.0 (23.0, 27.5)
* Missing*	*39 (1.3)*	*166 (6.7)*	*62 (2.2)*	*200 (8.4)*
Smoking				
No	1569 (55.2)	1227 (51.5)	1251 (55.2)	1059 (50.9)
Quit smoking before pregnancy	891 (31.4)	582 (24.4)	682 (30.1)	519 (24.9)
Smoked during pregnancy	380 (13.4)	575 (24.1)	335 (14.8)	503 (24.2)
* Missing*	*227 (7.4)*	*80 (3.2)*	*563 (19.9)*	*311 (13.0)*
Alcohol consumption				
No consumption < 3 months before pregnancy	626 (21.1)	291 (12.1)	529 (21.6)	247 (11.8)
Consumption < 3 months before pregnancy	1912 (64.4)	2113 (87.9)	1507 (61.5)	1854 (88.2)
Consumption during pregnancy	431 (14.5)	NA	413 (16.9)	NA
* Missing*	*98 (3.2)*	*60 (2.4)*	*382 (13.5)*	*291 (12.2)*
Parity				
Nulliparous	1952 (65.6)	1601 (66.3)	1596 (63.7)	1440 (65.7)
Multiparous	1022 (34.4)	812 (33.7)	911 (36.3)	751 (34.3)
* Missing*	*93 (3.0)*	*51 (2.1)*	*324 (11.4)*	*201 (8.4)*
Miscarriage in previous pregnancy				
No	2235 (79.3)	1809 (80.2)	1783 (79.8)	1581 (81.0)
Yes	585 (20.7)	447 (19.8)	451 (20.2)	372 (19.0)
* Missing*	*247 (8.1)*	*208 (8.4)*	*597 (21.1)*	*439 (18.4)*
Time to pregnancy, median (95% range), months ^e^	3.8 (0.0–67.9)	3.5 (0.0–59.9)	3.6 (0.0–65.3)	3.5 (0.0–58.1)
≤ 12 months	1964 (64.0)	1788 (72.6)	1650 (70.2)	1519 (73.3)
> 12 months^f^	554 (18.1)	433 (17.6)	436 (18.5)	349 (16.8)
ART leading to pregnancy^f^	290 (9.5)	230 (9.3)	265 (11.3)	204 (9.8)
Not pregnant^f^	259 (8.4)	13 (0.5)	NA	NA
* Missing*	*0 (0.0)*	*0 (0.0)*	*480 (17.0)*	*320 (13.4)*
Occurrence of miscarriage				
No miscarriage	2587 (92.1)	2283 (93.1)	2472 (87.3)	2142 (89.5)
Miscarriage	221 (7.9)	168 (6.9)	359 (12.7)	250 (10.5)
* Missing*	*259 (8.4)*	*13 (0.5)*	*0 (0.0)*	*0 (0.0)*
Timing of miscarriage, median (IQR), weeks	8.1 (7.0, 9.4)	8.4 (7.1, 9.4)	8.1 (7.0, 9.4)	8.3 (7.1, 9.4)
First trimester	203 (93.1)	155 (92.8)	328 (93.2)	231 (93.9)
Second trimester	16 (6.9)	12 (7.2)	25 (6.8)	15 (6.1)
* Missing*	*3 (1.4)*	*1 (0.6)*	*7 (1.9)*	*4 (1.6)*

*ART *assisted reproductive technology, *BMI *body mass index (calculated as weight in kilograms divided by height in meters squared), *NA *not applicable

^a^Total study population consisting of 3604 unique women from Rotterdam, the Netherlands with a total of 4036 episodes of which 3577 pregnancy episodes. Women were included in preconception and pregnancy between 2017 and 2021

^b^Study population of time to pregnancy consisting of 2882 unique women from Rotterdam, the Netherlands with a total of 3067 study episodes

^c^Study population of miscarriage consisting of 2528 unique women with a total of 2831 pregnancy episodes

^d^Included: European, German, Yugoslav, or Polish ethnicity

^e^Included: African; American, non-western; Asian, non-western; Chinese; Indonesian; American, western; Asian, western; Cape Verdean; Dutch Antilles; Moroccan; Oceanian; Surinamese; or Turkish

^f^Time to pregnancy in months was derived from pregnancy episodes with a natural conception

^g^Episodes with assisted reproductive technology (ART) leading to pregnancy (*n* = 290), episodes without pregnancy and use of ART (*n* = 7 of *n* = 259 episodes) and episodes without pregnancy and trying to become pregnant > 12 months (*n* = 217 of *n* = 259 episodes) were added to the infertile group (time to pregnancy > 12 months and ART) in the analysis

^h^Parity, miscarriage in previous pregnancy, time to pregnancy in months, occurrence of miscarriage, timing of miscarriage in weeks in men were derived from their partner

### Fecundability and infertility

**Fig. 2:**
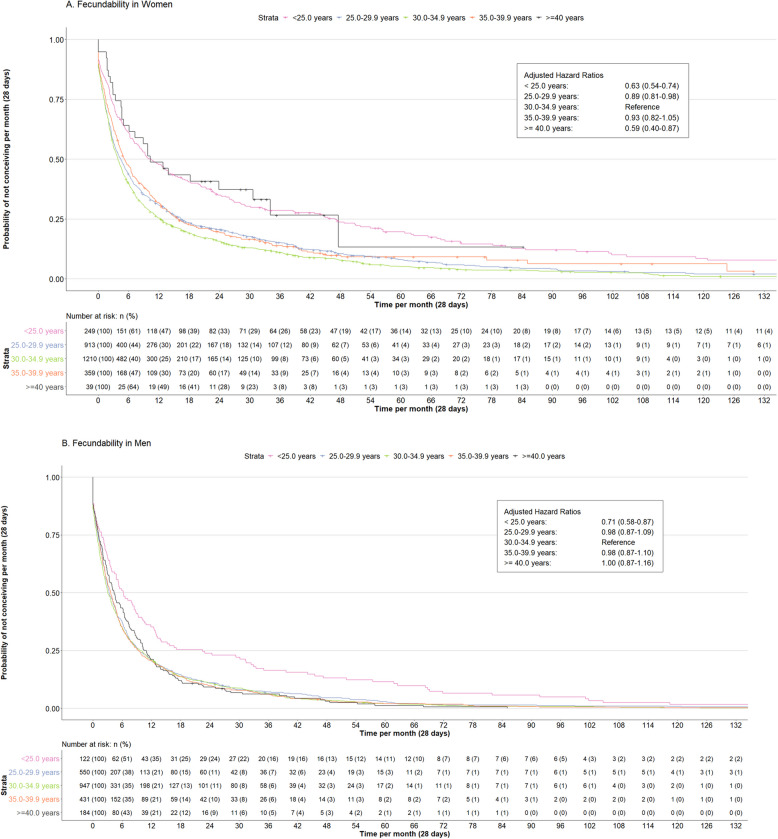
Associations of age at pursuing pregnancy of women and men and fecundability ratios. Fecundability ratios (with 95% confidence interval) associated with age at pursuing pregnancy in categories. Fecundability ratios were derived from the hazard ratios (HR) of the Cox model. The fecundability ratio of age on the categorical scale was calculated as follows: HR = (hazard rate (H(t)) of the different age categories)/(H(t) Age reference category: 30.0–34.9 years)). An HR < 1 indicates a lower fecundability as compared to the reference category. **A** Main analysis for women with adjustment for participants body mass index, ethnicity, education level, smoking, alcohol consumption and parity (Additional file 1: Tables S8–S9). Survival curve derived from unadjusted model. **B** Main analysis for men with adjustment for participants body mass index, ethnicity, education level, smoking and alcohol consumption (Additional file 1: Tables S8–S9). Survival curve derived from unadjusted model. Several sensitivity analyses were performed: excluding the top 5% observations of time to pregnancy correcting for the right-skewed distribution (Additional file 1: Table S10), including only first episodes of participants (Table S11), including partners age at pursuing pregnancy as a covariate (Additional file 1: Table S12), and including only the preconception cohort (Additional file 1: Table S13)

**Fig. 3:**
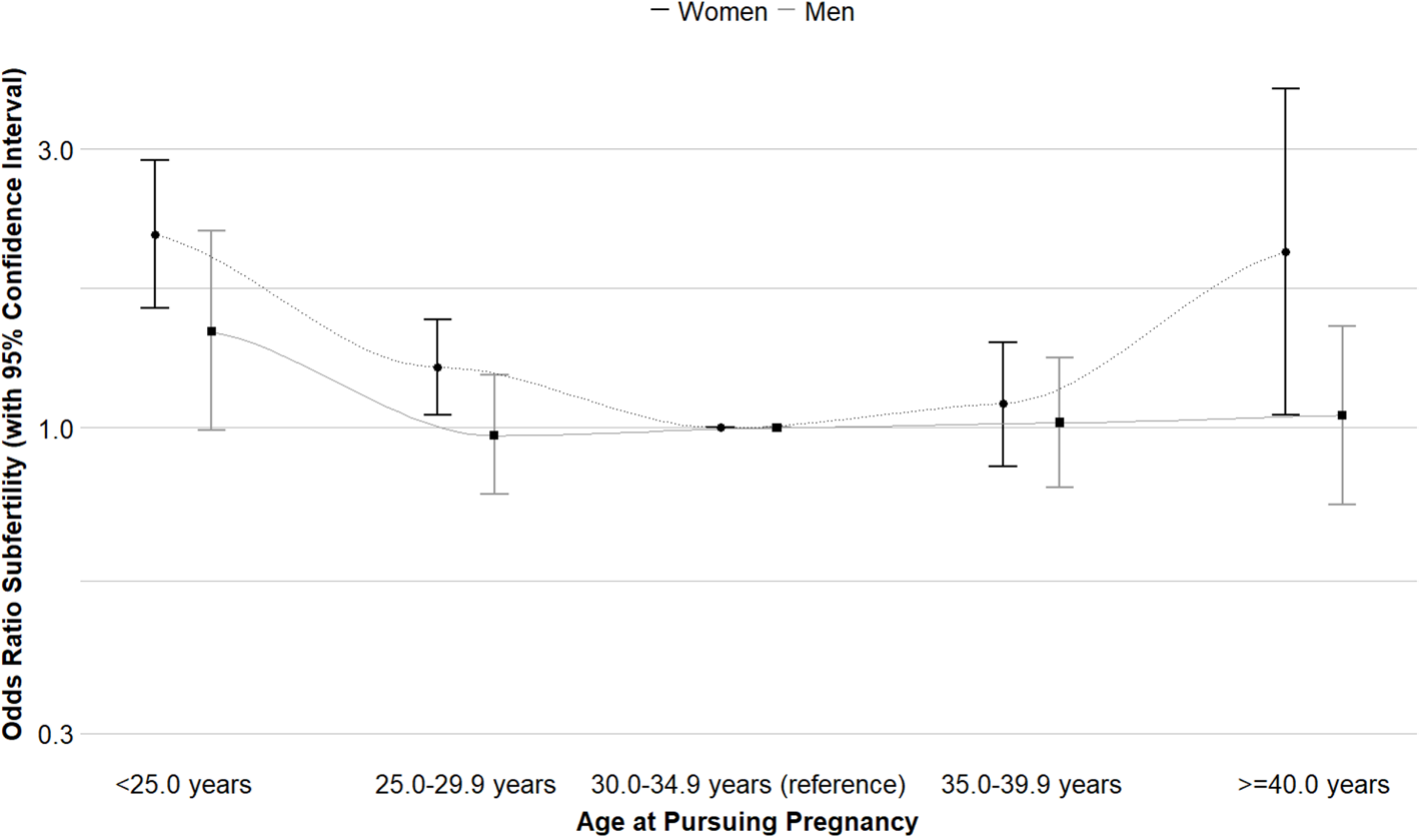
Associations of age at pursuing pregnancy of women and men and odds of infertility. Odds ratios (OR) (with 95% confidence interval) of infertility (time to pregnancy > 12 months and assisted reproductive technology) associated with age at pursuing pregnancy in categories. ORs were derived from the exponentiated coefficients of the logistic regression models. An OR > 1 indicates increased odds of infertility as compared to the reference category (30.0–34.9 years). Main analysis for women with adjustment for participants body mass index, ethnicity, education level, smoking, alcohol consumption and parity (Additional file 1: Tables S15–S16). Main analysis for men with adjustment for participants body mass index, ethnicity, education level, smoking and alcohol consumption (Additional file 1: Tables S15–S16). Several sensitivity analyses were performed, excluding episodes with assisted reproductive technology (Additional file 1: Table S17), excluding the top 5% observations of time to pregnancy correcting for the right-skewed distribution (Additional file 1: Table S18), including only first episodes of participants (Additional file 1: Table S19), including partners age at pursuing pregnancy as a covariate (Additional file 1: Table S20), and including only the preconception cohort (Additional file 1: Table S21)

Figure 2 shows that as compared to women aged 30.0–34.9 years, those aged < 25.0 years, 25.0–29.9 years, and ≥ 40.0 years had a lower fecundability (FR, 0.63 (95% confidence interval (CI), 0.54–0.74), FR, 0.89 (95% CI, 0.81–0.98) and FR, 0.59 (95% CI, 0.40–0.87), respectively). As compared to men aged 30.0–34.9 years, those aged < 25.0 years had lower fecundability (FR, 0.71 (95% CI, 0.58–0.87)), but not those aged 25.0–29.9, 35.0–39.9 and ≥ 40.0 years. Figure 3 shows that as compared to women aged 30.0–34.9 years, those aged < 25.0, 25.0–29.9 and ≥ 40.0 years had increased odds of infertility (odds ratio (OR) 2.14 (95% CI, 1.60–2.87); OR 1.27 (95% CI, 1.05–1.53) and OR 2.00 (95% CI, 1.05–3.80), respectively). We did not observe associations of age among men with the odds of infertility (Fig. 3). Analyses on combined age categories in both partners with fecundability and infertility showed no associations (Additional file 1: Table S14 and S22). Sensitivity analyses excluding women who underwent assisted reproductive technology, excluding women in the top 5% of the time to pregnancy observations, restricting analyses to only participants’ first episode or only the preconception cohort, or adjusting for partners’ age did not materially change the effect estimates (Additional file 1: Tables S10–S13 and S17–S21).

### Miscarriage

**Fig. 4:**
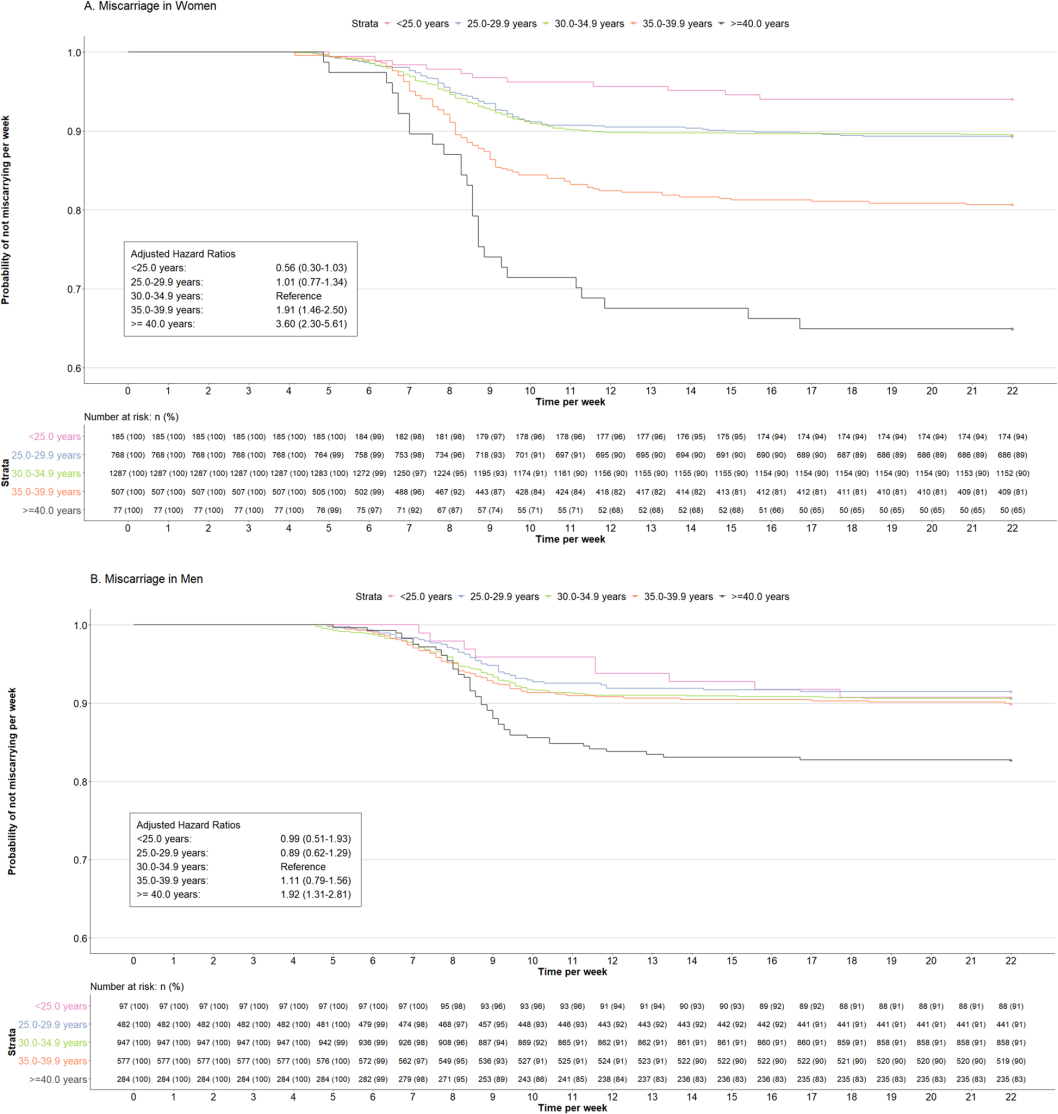
Associations of age at conception of women and men and hazard ratios for miscarriage. Hazard ratios (HR) (with 95% confidence interval) of miscarriage associated with age at conception in categories. HRs were derived from the Cox model. The HR of age on the categorical scale was calculated as follows: HR = (hazard rate (H(t)) of the different age categories)/(H(t) age reference category (30.0–34.9 years)). An HR > 1 indicates a higher probability of miscarriage per week as compared to the reference category. **A** Main analysis for women with adjustment for participants body mass index, ethnicity, education level, smoking, alcohol consumption, parity and history of miscarriage (Additional file 1: Tables S23–S24). Survival curve derived from unadjusted model. **B** Main analysis for men with adjustment for participants body mass index, ethnicity, education level, smoking and alcohol consumption (Additional file 1: Tables S23–S24). Survival curve derived from unadjusted model. Several sensitivity analyses were performed, excluding episodes with assisted reproductive technology (Additional file 1: Table S25), including only first episodes of participants (Additional file 1: Table S26), including partners age at conception as a covariate (Additional file 1: Table S27), and including only the preconception cohort (Additional file 1: Table S28)

**Fig. 5:**
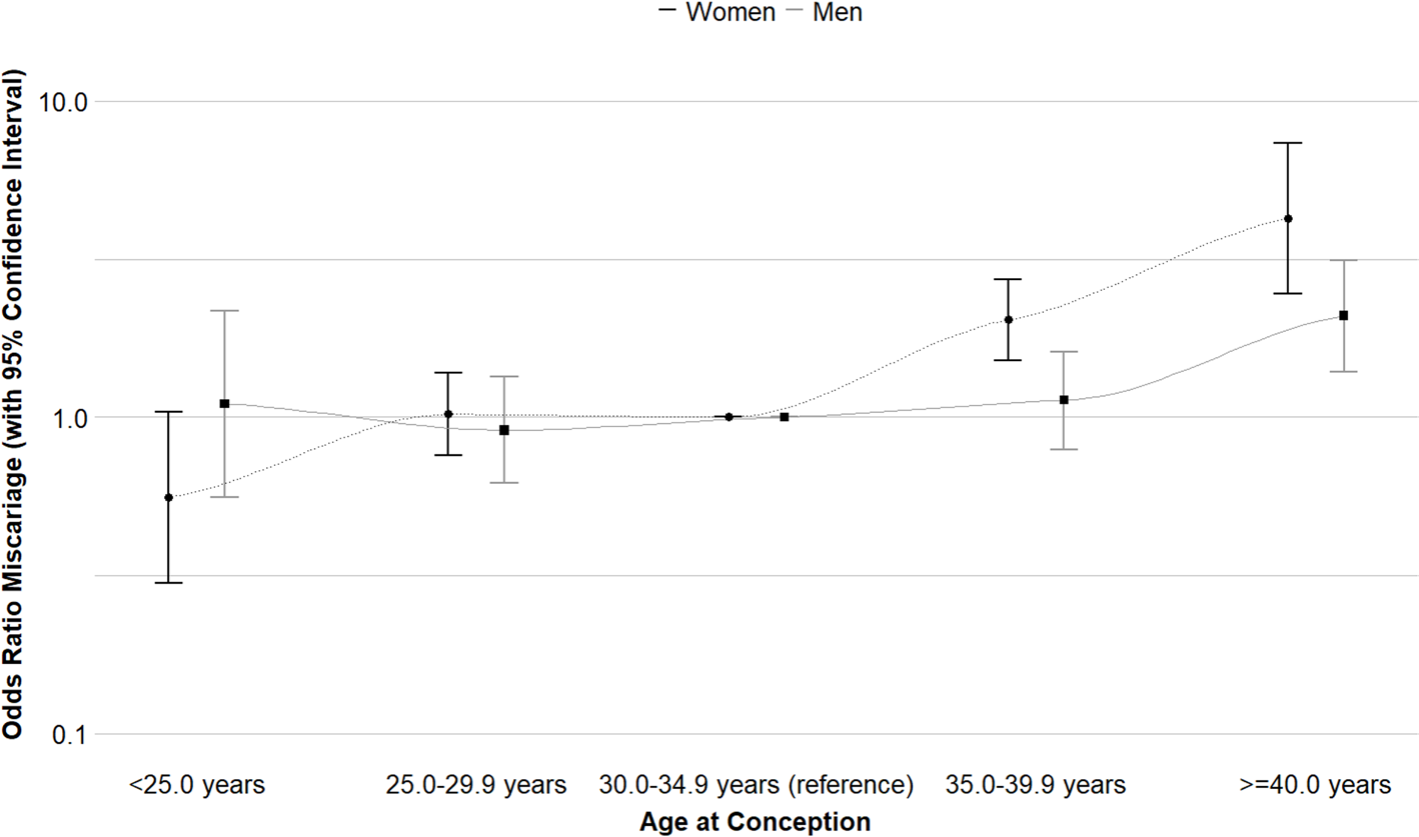
Associations of age at conception of women and men and odds of miscarriage. Odds ratios (OR) (with 95% confidence interval) of miscarriage associated with age at conception in categories. ORs were derived from the exponentiated coefficients of the logistic regression models. An OR > 1 indicates increased odds of miscarriage as compared to the reference category (30.0–34.9 years). Main analysis for women with adjustment for participants age, ethnicity, education level, smoking, alcohol consumption, parity and history of miscarriage (Additional file 1: Tables S30–S31). Main analysis for men with adjustment for participants age, ethnicity, education level, smoking and alcohol consumption (Additional file 1: Tables S30–S31). Several sensitivity analyses were performed, excluding episodes with assisted reproductive technology (Additional file 1: Table S32), including only first episodes of participants (Additional file 1: Table S33), and including partners age at conception as a covariate (Additional file 1: Table S34), and including only the preconception cohort (Additional file 1: Table S35)

Figure 4 shows that as compared to women aged 30.0–34.9 years at conception, those aged 35.0–39.9 years and ≥ 40.0 years had an increased risk of miscarriage (HR 1.91 (95% CI, 1.46–2.50) and HR, 3.60 (95% CI, 2.30–5.61), respectively). As compared to men aged 30.0–34.9 years at conception, those aged ≥ 40.0 years had an increased risk of miscarriage (HR, 1.92 (95% CI, 1.31–2.81)). Analyses on combined age categories in both partners showed that compared with both partners aged < 35 years at conception, both partners aged ≥ 35 years at conception increased the risk of miscarriage (HR, 2.18 (95% CI, 1.53–3.10)) (Additional file 1: Table S29). In line with these findings, Fig. 5 shows that as compared to women aged 30.0–34.9 years at conception, those aged 35.0–39.9 and ≥ 40.0 years had strongly increased odds of miscarriage (OR 2.03 (95% CI, 1.51–2.72) and 4.24 (95% CI, 2.45–7.36), respectively). As compared to men aged 30.0–34.9 years at conception, those aged ≥ 40.0 years had increased odds of miscarriage (OR, 2.09 (95% CI, 1.39–3.14)). Analyses on combined age categories in both partners showed that compared with both partners aged < 35 years at conception, both partners aged ≥ 35 years at conception had increased odds of miscarriage (OR, 2.33 (95% CI, 1.61–3.37)) (Additional file 1: Table S36). Sensitivity analyses excluding women who underwent assisted reproductive technology, restricting analyses to only participants’ first episode or only the preconception cohort, or adjusting for partners’ age did not materially change the effect estimates (Additional file 1: Tables S25–S28 and S32–S35).

## Discussion

In this population-based prospective cohort study from the preconception period until birth, we observed that the age at pursuing pregnancy of women outside the range of 30–40 years and of men below 25 years was associated with lower fecundability and infertility. Furthermore, we observed that the age at conception of women aged ≥ 35 years and of men aged ≥ 40 years was associated with an increased miscarriage risk. The range of 30.0–34.9 years for age at pursuing pregnancy and conception for women, 25.0–39.9 years for men, and < 35 years for both partners was associated with the lowest risk of experiencing adverse reproductive outcomes.

Older age at conception in women is a well-known and strong risk factor for infertility, miscarriage, pregnancy complications, and adverse birth outcomes [5, 10–19]. The associations of age with reduced fertility and risk of miscarriage might be present across the full reproductive age range and in both women and men [12, 13, 26–28]. However, while much is known about the adverse effects of advanced maternal age, there is limited evidence discussing the potential impact of younger age among women and the separate and combined effects of age among both women and men on fertility outcomes in the general population. Better knowledge about the age effects on fertility and early pregnancy outcomes could lead to better effective and balanced preventive strategies and counseling for couples.

We observed that women aged < 30 years and ≥ 40 years pursuing pregnancy had a lower fecundability and increased odds of infertility, even when excluding couples with assisted reproductive technology. Results from a retrospective cohort study among 61815 infertile women in Germany reported a lower pregnancy rate in women with increasing age [37]. A cohort study among 1924 couples in the Netherlands reported that women aged > 35 had lower fecundability as compared to women aged 30–35 years [12]. However, this was not a preconception but a pregnancy cohort study. A preconception cohort study among 2992 couples in the USA and Canada reported a lower fecundability among women aged ≥ 34 years as compared to women aged 21–24 years [13]. Thus, among women, older age seems to be associated with lower fertility. Both lifestyle and biological factors, such as body mass index and declining oocyte quantity and quality, may explain these associations [29, 38, 39]. However, in our study, the patterns of the associations were similar without and with adjustment for socio-demographic and lifestyle factors. Surprisingly, we observed that women aged < 30 years had lower fecundability. This is in contrast to previous literature, including studies in natural fertility populations, observing a higher fecundability among younger women [12, 13, 37, 40]. While residual confounding or selection may partly explain this association, another possible explanation could be the decline in fertility among younger cohorts [41, 42]. We also observed a lower fecundability among men aged < 25 years, strengthening this explanation. Furthermore, we hypothesize that this finding could be explained by less knowledge regarding reproduction and strategic family planning in younger couples as compared to older couples [43]. Furthermore, younger couples could engage in more lifestyle risk behavior, such as smoking or alcohol consumption, reducing their fecundability [44]. Based on this, we emphasize that age-specific preventive strategies for both younger and older couples are needed to achieve optimal counseling for women and men in all age groups [45].

The effect of the paternal age on fertility outcomes is not well known. We observed that men aged < 25 years had a lower fecundability. In contrast to our findings, a previous cohort study among 1924 couples in the Netherlands found no association between the age of men and fecundability [12]. We did not observe an association of the age of men with infertility. A pregnancy cohort study among 8515 pregnancies in the UK did report higher odds of infertility among men aged 30–34, 35–39, and ≥ 40 years as compared to men aged < 25 years [46]. When we combined the age of women and men in one model, we did not observe associations of age with fecundability or infertility. This is probably due to small sample size per age group. Thus, in this large European population-based cohort, both younger and older age at pursuing pregnancy among women and older age among men was associated with lower fecundability and infertility.

Older age at conception is a well-known risk factor for early pregnancy complications, including miscarriage [13–16]. We observed that age at conception of ≥ 35 years in women was associated with a higher probability of miscarriage per week and higher odds of miscarriage. An observational follow-up study among 88373 pregnancies in Denmark observed a higher miscarriage risk in women aged ≥ 30 years [47]. However, this was not a prospective preconception cohort study but a follow-up study of a pregnancy cohort. In line with our study, a population-based cohort among 525604 pregnant women in Sweden and a multisite retrospective cohort study among 3174 pregnancies of European women found that women aged ≥ 35 years had an increased risk of miscarriage [48, 49]. Among men, we observed that men aged ≥ 40 years had increased odds of miscarriage. A meta-analysis and a systematic review found that age of ≥ 40 years in men was associated with miscarriage [27, 50]. A population-based cohort among 525604 pregnant women in Sweden and a prospective cohort among 5121 women in the USA observed an association of age of ≥ 35 years at conception in men with miscarriage [48, 51]. When we combined the age of women and men in one model, we observed that an age of ≥ 35 years at conception in women and men increased the probability of miscarriage per week and the overall risk of miscarriage. This finding is in line with results from a retrospective multisite population-based cohort study among 3174 pregnancies of European women [49]. The occurrence of miscarriage is dependent on different factors, including lifestyle behavior and socio-demographic factors [14]. However, in our analysis, the observed associations did not change when correcting for these factors. Furthermore, ectopic pregnancies and terminations were seen as “no miscarriage” due to the different pathophysiological mechanisms involved. When we excluded the *n* = 30 ectopic pregnancies, we did observe similar effect estimates for the miscarriage analyses. Altogether, results from our and previous studies strongly suggest that older age among women and men is associated with increased risks of miscarriage.

The potential biological mechanisms underlying associations of age with fertility and early pregnancy outcomes seem to include various biological processes such as lower gamete quality and a decline in oocyte reserve and endometrium receptivity [7–9, 24, 25]. Among women, older age is associated with a reduced number of oocytes and a decline in their quality [38]. This negatively impacts fertilization and embryo development and subsequently increases the risk of chromosomal abnormalities leading to miscarriage [52–54]. Older age among women also decreases endometrium receptivity as a consequence of declining endometrium thickness, alterations in endometrial epithelium, and microscopic tissue fibrosis [55]. This affects implantation rate and fertility [56]. Among older men, testicular function declines due to lower levels of testosterone and gonadotropins, leading to lower sperm volume, motility, and morphology, and subsequently lower fertility [57, 58]. Sperm cells of men with older age have more DNA fragmentation [26, 59]. DNA fragmentation most likely occurs due to a combination of an increase in reactive oxygen species, a decrease in antioxidant capacity, and decreased function of DNA repair mechanisms [27, 60]. In clinical populations, sperm cell DNA fragmentation is associated with recurrent pregnancy loss [50, 61, 62].

We also observed that the younger age of both women and men was associated with increased risks of infertility [43, 44]. As discussed previously, human reproductive health seems to be declining in the past century [41, 42, 63]. We speculate that our findings may reflect biological or environmental factors adversely affecting fertility at earlier ages, such as endocrine-disrupting exposures or lifestyle-related risks [63]. A recent study has highlighted such mechanisms as potential contributors to declining human fertility across age groups [63]. Therefore, these findings should be interpreted carefully and suggest that further research is needed. Other possible explanations for lower fertility in younger age groups could be poor fertility knowledge and unawareness regarding the importance of preconception health, engaging in lifestyle risk behavior, and less strategic family planning [64–66]. This could possibly lead to less effective reproduction and thus a longer time to conceive among younger couples.

Our results suggest that age across the full reproductive age range among women and men in the general population is an important risk factor for decreased fertility and adverse (early) pregnancy outcomes. In western countries, age at childbearing is increasing, leading to infertility and increased miscarriage risk. This may have a great impact on individuals and couples, as well as on health care costs related to fertility and pregnancy care. Being aware of the effect of age among women and men on fertility outcomes and health risks for mother and child could motivate couples to start family planning at an optimal age. This could lead to effective and balanced preventive strategies focused on both women and men, taking into account different cultural, social, and economic factors.

### Methodological considerations

To the best of our knowledge, this was one of the population-based prospective cohorts assessing the associations of age among women and men separately and combined across the full reproductive age range with fertility and early pregnancy outcomes. Strengths were the large sample size from the preconception or early pregnancy period onwards, inclusion of men, and the use of two complementary measures of age: age at pursuing pregnancy, a measure which is not used very often, with fertility outcomes, and age at conception with miscarriage outcomes. However, several limitations should be discussed. First, non-response analyses showed that the included and excluded populations differed, which could limit the generalizability of the results to other populations. The excluded population was on average younger, which may have led to an underestimation of the associations between younger age and reproductive outcomes. Second, although the design of this cohort aimed for inclusion during the preconception period or early pregnancy, 33.2% of the participants were enrolled before conception. This is particularly relevant for time to pregnancy and miscarriage as outcomes since both occur around the time of conception or in early pregnancy. Although the baseline characteristics of participants included in preconception and early pregnancy periods were similar, we observed that the proportion of miscarriages was higher in the preconception cohort. This might have led to a higher reported miscarriage rate if all participants were included during the preconception period, since up to 30% of conceptions end in very early pregnancy loss [67]. Thus, including more women in the preconception period could result in a stronger association of age with miscarriage risk. Also, we observed that the median time to pregnancy was longer in the preconception cohort, although both cohorts showed a similar 95% range. However, time to pregnancy may be misclassified for some participants, particularly those enrolled during early pregnancy, because they may inaccurately recall the start date of pursuing pregnancy [68, 69]. This may partially explain differences in the time to pregnancy distribution between cohorts. To address this, we conducted a sensitivity analysis restricted to the preconceptionally included participants. We observed that associations of age with time to pregnancy and miscarriage within the preconception cohort were similar to those of the full cohort. Consequently, time to pregnancy estimates based on the total cohort may underestimate the associations of age with fecundability and infertility. Third, the date at which couples actively pursued pregnancy was self-reported and could have been misinterpreted in our questionnaires, leading to misclassification. To take account for this, we used the date of pursuing pregnancy combined with the date of refraining from contraceptives to assess age at pursuing pregnancy. Fourth, the accuracy of time to pregnancy duration and duration of pursuing pregnancy may have been biased by the potentially retrospectively answered questionnaires. To address this issue, time to pregnancy was reconfirmed during the first trimester visit and the duration of pursuing pregnancy in the yearly preconception questionnaires. Fifth, we used months rather than menstrual cycles to estimate time to pregnancy, which may have introduced misclassification, particularly for women with irregular menstrual cycles. This could have led to an overestimation of the number of conception attempts. However, infertility is clinically defined by months instead of menstrual cycles, and the use of months instead of menstrual cycles for estimating fecundability is common in population-based studies [33]. While our approach might have overestimated the time to pregnancy, the use of a clinical definition of infertility allows for the translation of the results into clinical care. Sixth, early pregnancy losses could have been misclassified as non-conceptions, especially if the loss occurred before pregnancy recognition. This may have led to an underestimation of conception rates, particularly in older age groups where early pregnancy losses are more common [70]. As a result, the impact of age on fecundability might be slightly overestimated, since unrecognized early pregnancy losses in older women would lead to an incorrect prolonged time to pregnancy. Finally, although we adjusted for multiple confounders, residual confounding might still be an issue because of the observational nature of the study.

## Conclusions

In this population-based prospective cohort study from the preconception period until birth, we observed a U-shaped relationship of female and male age with reduced fertility. Women aged younger than 30 years and older than 40 years and men aged younger than 25 years have a higher risk of reduced fertility. Women aged older than 35 years, men aged older than 40 years, and couples with both partners aged older than 35 years have a higher miscarriage risk. Strategies focusing on both women and men to optimize the age at which couples start family planning could have a great individual and public health impact.

## Supplementary Information

Additional file 1.

## Data Availability

Data are available upon request to the corresponding author. Unrestricted data sharing is not allowed due to ethical consent and privacy restrictions.

## Abbreviations

BMI: Body mass index
CI: Confidence interval
DAG: Directed acyclic graph
FR: Fecundability ratio
HR: Hazard ratio
IVF: In vitro fertilization
IUI: Intrauterine insemination
OR: Odds ratio
