# Supplementary material for: Age among women and men, time to pregnancy and risk of miscarriage
Source: BMC Med. 2025 Nov 17;23:639. doi: 10.1186/s12916-025-04462-8 (PMC12625503; doi:10.1186/s12916-025-04462-8)
Supplement: Supplementary file 1 — Additional file 1. [file 12916_2025_4462_MOESM1_ESM.docx]

**Additional file 1**

**Age among Women and Men, Time to Pregnancy**

**and Risk of Miscarriage**

Aline J. Boxem MD^1,2^, Sophia M. Blaauwendraad MD^1,2^, Annemarie G. M.G. J. Mulders MD, PhD^3^, Eline L. Bekkers MSc^1^, Romy Gaillard MD, PhD^1,2^, Vincent W.V. Jaddoe MD, PhD^1,2^

^1^ The Generation R Study Group, Erasmus MC, University Medical Centre, Rotterdam, the Netherlands.

^2^ Department of Paediatrics, Erasmus MC, University Medical Centre, Rotterdam, the Netherlands.

^3^ Department of Obstetrics and Gynaecology, Erasmus MC, University Medical Centre, Rotterdam, the Netherlands.

**Corresponding author**

Vincent W.V. Jaddoe, The Generation R Study Group (Na29-15), Erasmus MC, University Medical Centre, P.O. Box 2040, 3000 CA Rotterdam, the Netherlands. E-mail: [v.jaddoe@erasmusmc.nl](mailto:v.jaddoe@erasmusmc.nl).

**TABLE OF CONTENT**

[SUPPLEMENTAL METHODS 6](#_Toc211520751)

[Calculation of Fecundability Ratio on the Continuous and Categorical Scale 7](#_Toc211520752)

[SUPPLEMENTAL FIGURES 8](#_Toc211520753)

[Figure S1. Description of Study Design by Inclusion Moment. 9](#_Toc211520754)

[Figure S2. Directed Acyclic Graph of Age and Time to Pregnancy. 10](#_Toc211520755)

[Figure S3. Directed Acyclic Graph of Age and the Odds of Miscarriage. 10](#_Toc211520756)

[SUPPLEMENTAL TABLES 11](#_Toc211520757)

[Table S1. Non-Response Analysis of Participants Included and Excluded From the Study Populations. 12](#_Toc211520758)

[Table S2. Non-Response Analysis of Episodes Included and Excluded from the Study Populations. 15](#_Toc211520759)

[Table S3. Population Characteristics stratified per Group of Age at Pursuing Pregnancy of Female Episodes. 18](#_Toc211520760)

[Table S4. Population Characteristics stratified per Group of Age at Pursuing Pregnancy of Male Episodes. 20](#_Toc211520761)

[Table S5. Population Characteristics stratified per Group of Age at Conception of Female Episodes. 22](#_Toc211520762)

[Table S6. Population Characteristics stratified per Group of Age at Conception of Male Episodes. 24](#_Toc211520763)

[Table S7. Population Characteristics stratified per Inclusion Moment. 26](#_Toc211520764)

[Supplemental Tables of Associations of Age among Women and Men with Fecundability Ratios. 28](#_Toc211520765)

[Table S8. Associations of Age among Women and Men with Fecundability Ratios, Basic Model. 28](#_Toc211520766)

[Table S9. Associations of Age among Women and Men with Fecundability Ratios, Adjusted Model. 29](#_Toc211520767)

[Table S10. Associations of Age among Women and Men with Fecundability Ratios, Adjusted Model excluding Top 5% of Time to Pregnancy. 30](#_Toc211520768)

[Table S11. Associations of Age among Women and Men with Fecundability Ratios, Adjusted Model including only First Episodes. 31](#_Toc211520769)

[Table S12. Associations of Age among Women and Men with Fecundability Ratios, Adjusted Model including Age of Partner. 32](#_Toc211520770)

[Table S13. Associations of Age among Women and Men with Fecundability Ratios, Adjusted Model including only Preconceptionally Included Episodes. 33](#_Toc211520771)

[Table S14. Associations of Joint Age of Women and Men with Fecundability Ratios, Adjusted Model. 34](#_Toc211520772)

[Supplemental Tables of Associations of Age among Women and Men with Odds of Infertility. 35](#_Toc211520773)

[Table S15. Associations of Age among Women and Men with Odds of Infertility, Basic Model. 35](#_Toc211520774)

[Table S16. Associations of Age among Women and Men with Odds of Infertility, Adjusted Model. 36](#_Toc211520775)

[Table S17. Associations of Age among Women and Men with Odds of Infertility, Adjusted Model excluding Couples undergoing Assisted Reproductive Technology. 37](#_Toc211520776)

[Table S18. Associations of Age among Women and Men with Odds of Infertility, Adjusted Model excluding Top 5% of Time to Pregnancy. 38](#_Toc211520777)

[Table S19. Associations of Age among Women and Men with Odds of Infertility, Adjusted Model including only First Episodes. 39](#_Toc211520778)

[Table S20. Associations of Age among Women and Men with Odds of Infertility, Adjusted Model including Age of Partner. 40](#_Toc211520779)

[Table S21. Associations of Age among Women and Men with Odds of Infertility, Adjusted Model including only Preconceptionally Included Episodes. 41](#_Toc211520780)

[Table S22. Associations of Joint Age of Women and Men with Odds of Infertility, Adjusted Model. 42](#_Toc211520781)

[Supplemental Tables of Associations of Age among Women and Men with Hazard Ratios of Miscarriage. 43](#_Toc211520782)

[Table S23. Associations of Age among Women and Men with Hazard Ratios of Miscarriage, Basic Model. 43](#_Toc211520783)

[Table S24. Associations of Age among Women and Men with Hazard Ratios of Miscarriage, Adjusted Model. 44](#_Toc211520784)

[Table S25. Associations of Age among Women and Men with Hazard Ratios of Miscarriage, Adjusted Model excluding Couples undergoing Assisted Reproductive Technology. 45](#_Toc211520785)

[Table S26. Associations of Age among Women and Men with Hazard Ratios of Miscarriage, Adjusted Model including only First Episodes. 46](#_Toc211520786)

[Table S27. Associations of Age among Women and Men with Hazard Ratios of Miscarriage, Adjusted Model including Age of Partner. 47](#_Toc211520787)

[Table S28. Associations of Age among Women and Men with Hazard Ratios of Miscarriage, Adjusted Model including only Preconceptionally Included Episodes. 48](#_Toc211520788)

[Table S29. Associations of Joint Age of Women and Men with Hazard Ratios of Miscarriage, Adjusted Model. 49](#_Toc211520789)

[Supplemental Tables of Associations of Age among Women and Men with Odds of Miscarriage. 50](#_Toc211520790)

[Table S30. Associations of Age among Women and Men with Odds of Miscarriage, Basic Model. 50](#_Toc211520791)

[Table S31. Associations of Age among Women and Men with Odds of Miscarriage, Adjusted Model. 51](#_Toc211520792)

[Table S32. Associations of Age among Women and Men with Odds of Miscarriage, Adjusted Model excluding Couples undergoing Assisted Reproductive Technology. 52](#_Toc211520793)

[Table S33. Associations of Age among Women and Men with Odds of Miscarriage, Adjusted Model including only First Episodes. 53](#_Toc211520794)

[Table S34. Associations of Age among Women and Men with Odds of Miscarriage, Adjusted Model including Age of Partner. 54](#_Toc211520795)

[Table S35. Associations of Age among Women and Men with Odds of Miscarriage, Adjusted Model including only Preconceptionally Included Episodes. 55](#_Toc211520796)

[Table S36. Associations of Joint Age of Women and Men with Odds of Miscarriage, Adjusted Model. 56](#_Toc211520797)

# SUPPLEMENTAL METHODS

## Calculation of Fecundability Ratio on the Continuous and Categorical Scale

The Hazard Ratio (HR) of age was calculated by dividing the hazard rate of conceiving within one month with one unit increase or decrease in age by the hazard rate of the mean age:

HR = (H(t) ± 1 unit age) / (H(t) mean age),

or, dividing the hazard rate of conceiving within one month of the different categories of age by the hazard rate of the reference category of age:

HR = (H(t) ± age category) / (H(t) age reference category).

A HR < 1 indicates a lower fecundability per unit increase or decrease in age or as compared to the reference category of age.

# SUPPLEMENTAL FIGURES

## **Figure S1.** Description of Study Design by Inclusion Moment.


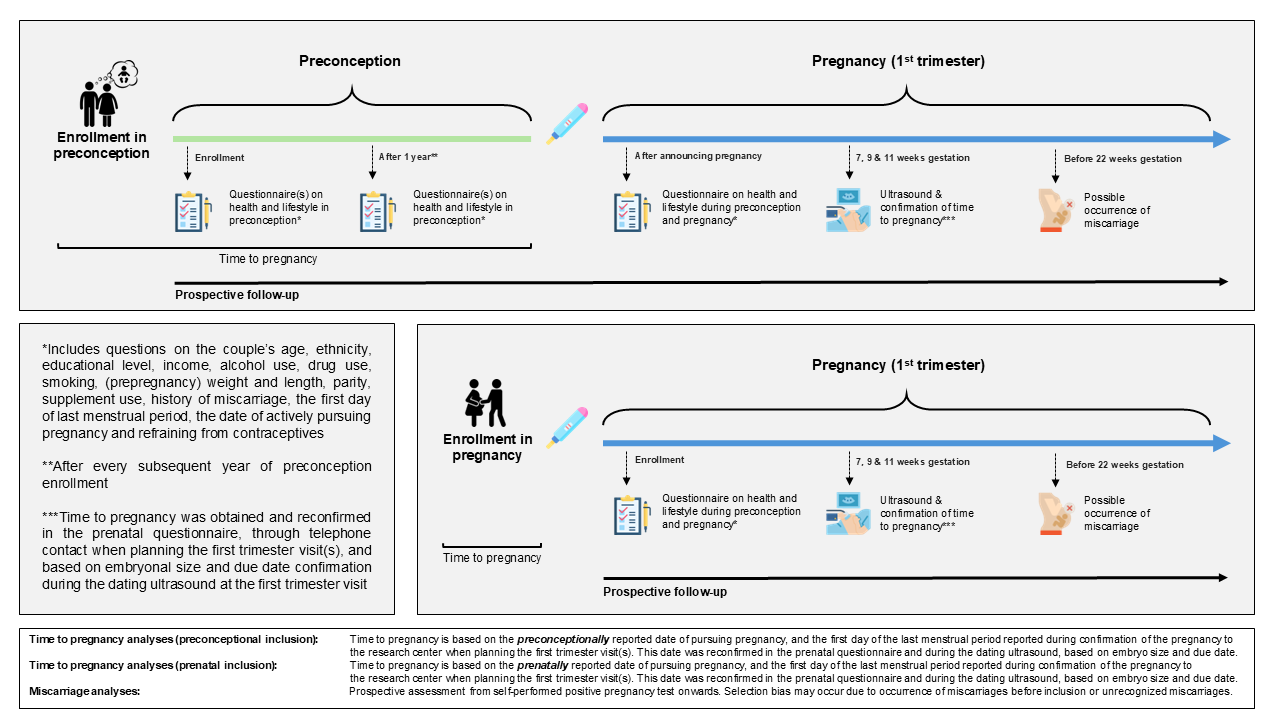


## Figure S2. Directed Acyclic Graph of Age and Time to Pregnancy.

**
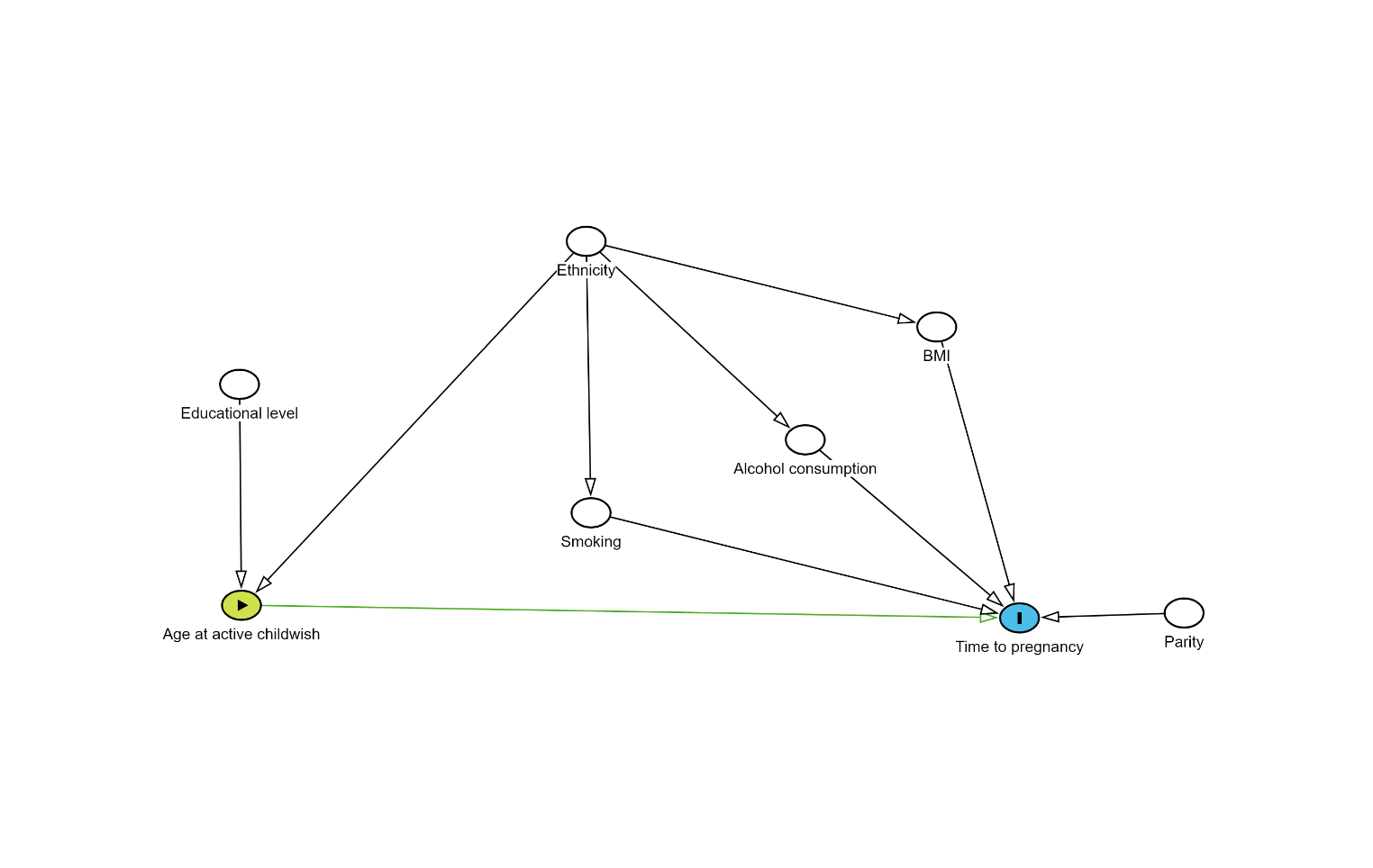
**

## Figure S3. Directed Acyclic Graph of Age and the Odds of Miscarriage.


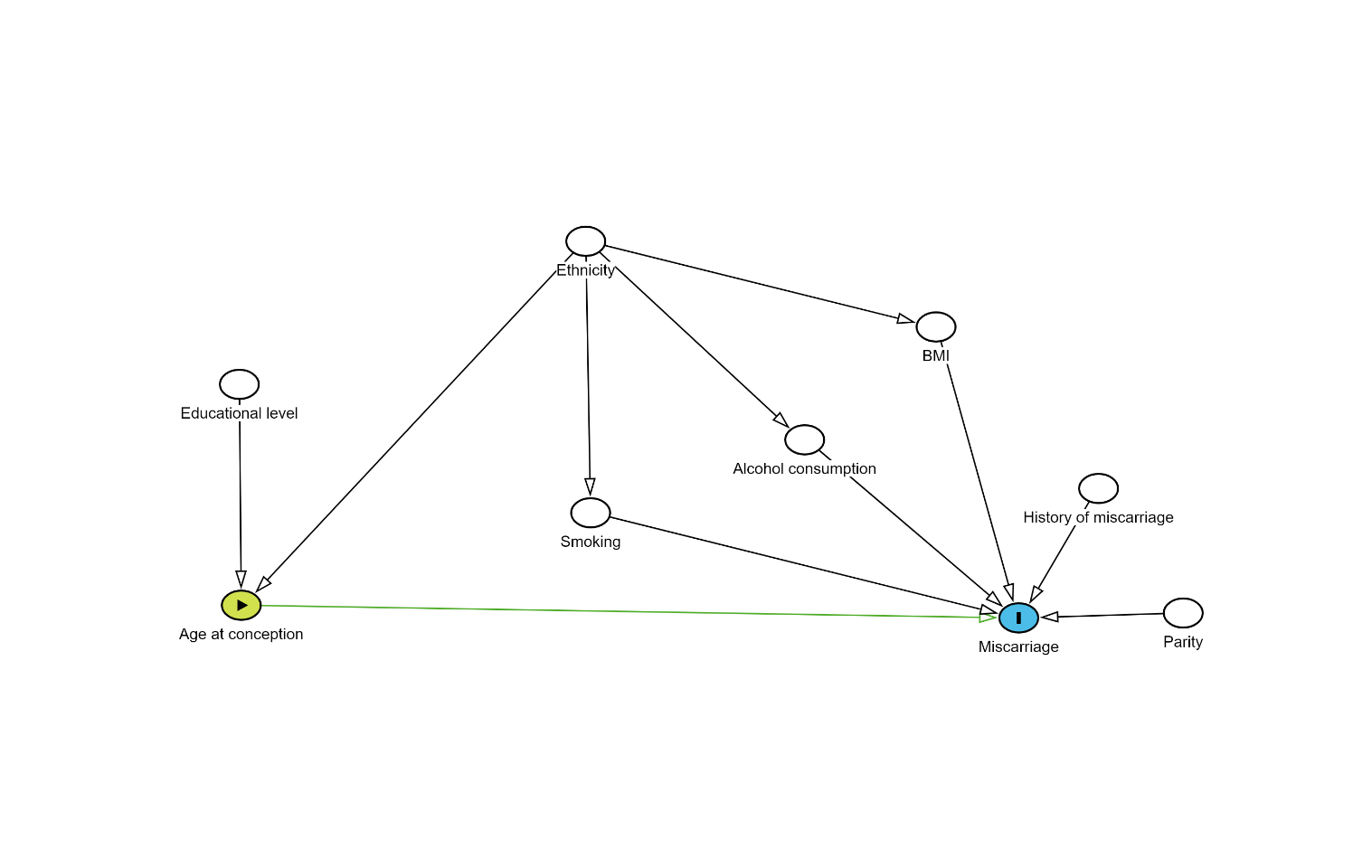


# SUPPLEMENTAL TABLES

## Table S1. Non-Response Analysis of Participants Included and Excluded From the Study Populations.

|  | **Women** | | | **Men** | | |
| --- | --- | --- | --- | --- | --- | --- |
|  | **Participants, No. (%)^a,b^** | | | **Participants, No. (%)^a,b^** | | |
|  | Non-responders | Responders | p-value | Non-responders | Responders | p-value |
|  | N=225 | N=3,217 |  | N=120 | N=2,496 |  |
| Age at pursuing pregnancy, median (IQR), y | 28.0 [28.0-28.0] | 30.7 [28.2, 33.2] | *0.74* | NA | 32.1 [29.4, 35.3] | *NA* |
| <25.0 | 0 (0.0) | 257 (9.1) |  | 0 (0.0) | 123 (5.5) |  |
| 25.0-29.9 | 1 (100.0) | 942 (33.3) |  | 0 (0.0) | 557 (24.9) |  |
| 30.0-34.9 | 0 (0.0) | 1222 (43.2) |  | 0 (0.0) | 945 (42.2) |  |
| 35.0-39.9 | 0 (0.0) | 368 (13.0) |  | 0 (0.0) | 433 (19.3) |  |
| ≥40.0 | 0 (0.0) | 39 (1.4) |  | 0 (0.0) | 181 (8.1) |  |
| *Missing* | *224 (99.6)* | *389 (12.1)* |  | *120 (100.0)* | *257 (10.3)* |  |
| Age at conception, median (IQR), y | 28.6 [24.9, 32.8] | 31.3 [28.6, 34.2] | *<0.01* | 31.4 [27.7, 35.6] | 33.1 [30.0, 36.4] | *<0.01* |
| <25.0 | 58 (25.9) | 220 (7.4) |  | 17 (14.2) | 105 (4.3) |  |
| 25.0-29.9 | 75 (33.5) | 864 (29.1) |  | 36 (30.0) | 516 (21.0) |  |
| 30.0-34.9 | 56 (25.0) | 1299 (43.7) |  | 33 (27.5) | 991 (40.3) |  |
| 35.0-39.9 | 31 (13.8) | 511 (17.2) |  | 28 (23.3) | 570 (23.2) |  |
| ≥40.0 | 4 (1.8) | 76 (2.6) |  | 6 (5.0) | 280 (11.4) |  |
| *Missing* | *1 (0.4)* | *247 (7.7)* |  | *0 (0.0)* | *34 (1.4)* |  |
| Ethnicity |  |  | *<0.01* |  |  | *<0.01* |
| Dutch | 14 (20.3) | 1791 (59.7) |  | 13 (31.7) | 1490 (63.9) |  |
| European^c^ | 9 (13.0) | 311 (10.4) |  | 4 (9.8) | 192 (8.2) |  |
| Non-European^d^ | 46 (66.7) | 898 (29.9) |  | 24 (58.5) | 649 (27.8) |  |
| *Missing* | *156 (69.3)* | *217 (6.7)* |  | *79 (65.8)* | *165 (6.6)* |  |
| Educational level |  |  | *<0.01* |  |  | *0.01* |
| No, primary, or secondary education finished | 38 (56.7) | 915 (30.7) |  | 23 (57.5) | 869 (37.0) |  |
| Higher education finished | 29 (43.3) | 2065 (69.3) |  | 17 (42.5) | 1477 (63.0) |  |
| *Missing* | *158 (70.2)* | *237 (7.4)* |  | *80 (66.7)* | *150 (6.0)* |  |
| BMI, median (IQR), kg/m^2^ | 24.3 [21.5, 28.1] | 23.6 [21.3, 26.9] | *0.22* | 26.1 [23.6, 28.7] | 25.0 [23.0, 27.5] | *0.31* |
| *Missing* | *141 (62.7)* | *60 (1.9)* |  | *32 (26.7)* | *213 (8.5)* |  |
| Smoking |  |  | *<0.01* |  |  | *0.62* |
| No | 14 (32.6) | 1583 (55.1) |  | 8 (47.1) | 1130 (50.5) |  |
| No, quit smoking before pregnancy | 7 (16.3) | 881 (30.7) |  | 3 (17.6) | 538 (24.0) |  |
| Yes, smoked during pregnancy | 22 (51.2) | 408 (14.2) |  | 6 (35.3) | 570 (25.5) |  |
| *Missing* | *182 (80.9)* | *345 (10.7)* |  | *103 (85.8)* | *258 (10.3)* |  |
| Alcohol consumption |  |  | *<0.01* |  |  | *0.45* |
| No consumption < 3 months before pregnancy | 27 (50.9) | 645 (22.4) |  | 4 (21.1) | 285 (12.6) |  |
| Consumption < 3 months before pregnancy | 15 (28.3) | 1814 (63.0) |  | 15 (78.9) | 1970 (87.4) |  |
| Consumption during pregnancy | 11 (20.8) | 422 (14.6) |  | NA | NA |  |
| *Missing* | *172 (76.4)* | *336 (10.4)* |  | *101 (84.2)* | *241 (9.7)* |  |
| Parity |  |  | *0.18* |  |  | *1.00* |
| Nulliparous | 17 (54.8) | 1949 (67.8) |  | 13 (68.4)^f^ | 1584 (69.1) ^f^ |  |
| Multiparous | 14 (45.2) | 926 (32.2) |  | 6 (31.6) ^f^ | 709 (30.9) ^f^ |  |
| *Missing* | *194 (86.2)* | *342 (10.6)* |  | *101 (84.2) ^f^* | *203 (8.1) ^f^* |  |
| Miscarriage in previous pregnancy |  |  | *0.91* |  |  | *0.32* |
| No | 25 (83.3) | 2303 (80.9) |  | 16 (94.1) ^f^ | 1858 (81.9) ^f^ |  |
| Yes | 5 (16.7) | 545 (19.1) |  | 1 (5.9) ^f^ | 411 (18.1) ^f^ |  |
| *Missing* | *195 (86.7)* | *369 (11.5)* |  | *103 (85.8)* ^f^ | *227 (9.1)* ^f^ |  |
| Time to pregnancy in months, median (95% range), mo.^e^ | NA | 3.5 [0.0-67.8] | *NA* | NA ^f^ | 3.1 [0.0-58.6] ^f^ | *NA* |
| *≤*12 months | 0 (0.0) | 1842 (64.9) |  | 0 (0.0) ^f^ | 1670 (74.4) ^f^ |  |
| >12 months | 0 (0.0) | 474 (16.7) |  | 0 (0.0) ^f^ | 355 (15.8) ^f^ |  |
| ART leading to pregnancy | 7 (87.5) | 276 (9.7) |  | 1 (100.0) ^f^ | 213 (9.5) ^f^ |  |
| Not pregnant | 1 (12.5) | 247 (8.7) |  | 0 (0.0) ^f^ | 8 (0.4) ^f^ |  |
| *Missing* | *217 (96.4)* | *378 (11.8)* |  | *119 (99.2) ^f^* | *250 (10.0) ^f^* |  |
| Occurrence of miscarriage |  |  | *NA* |  |  | *NA* |
| No miscarriage | 224 (100.0) | 2673 (90.0) |  | 120 (100.0) ^f^ | 2277 (91.5) ^f^ |  |
| Miscarriage | 0 (0.0) | 297 (10.0) |  | 0 (0.0) ^f^ | 211 (8.5) ^f^ |  |
| *Missing* | *1 (0.4)* | *247 (7.7)* |  | *0 (0.0) ^f^* | *8 (0.3) ^f^* |  |
| Timing of miscarriage, median (IQR), wk. | NA | 8.3 [7.1, 9.4] | *NA* | NA ^f^ | 8.4 [7.1, 9.4] ^f^ | *NA* |
| First trimester | 0 (0.0) | 272 (93.5) |  | 0 (0.0) ^f^ | 196 (94.2) ^f^ |  |
| Second trimester | 0 (0.0) | 20 (6.5) |  | 0 (0.0) ^f^ | 12 (5.8) ^f^ |  |
| *Missing* | *225 (100.0)* | *6 (2.0)* |  | *120 (100.0) ^f^* | *3 (1.4) ^f^* |  |
| Abbreviations: ART, assisted reproductive technology; BMI, body mass index (calculated as weight in kilograms divided by height in meters squared); NA, not applicable.  Women were included in preconception and pregnancy between 2017 and 2021. Values are presented as median (IQR), median (95% range) or number of participants (valid %).   1. Study population of time to pregnancy and miscarriage consisting of 3,217 unique women and 2,496 unique men from Rotterdam, the Netherlands. 2. Non-responders consisted of 225 unique women and 120 unique men from Rotterdam, the Netherlands. 3. Included: European, German, Yugoslav, or Polish ethnicity. 4. Included: African; American, non-western; Asian, non-western; Chinese; Indonesian; American, western; Asian, western; Cape Verdean; Dutch Antilles; Moroccan; Oceanian; Surinamese, or Turkish ethnicity. 5. Time to pregnancy in months was derived from pregnancy episodes with a natural conception. 6. Parity, miscarriage in previous pregnancy, time to pregnancy in months, occurrence of miscarriage, timing of miscarriage in weeks in men were derived from their partner. | | | | | | |

## Table S2. Non-Response Analysis of Episodes Included and Excluded from the Study Populations.

|  | **Women** | | | **Men** | | |
| --- | --- | --- | --- | --- | --- | --- |
|  | **Episodes, No. (%)^a,b^** | | | **Episodes, No. (%)^a,b^** | | |
|  | Non-responders | Responders | p-value | Non-responders | Responders | p-value |
|  | N=263 | N=3,569 |  | N=147 | N=2,796 |  |
| Age at pursuing pregnancy, median (IQR), y | 28.0 [28.0, 28.0] | 30.8 [28.2, 33.3] | *0.73* | NA | 32.1 [29.4, 35.4] | *NA* |
| <25.0 | 0 (0.0) | 277 (9.0) |  | 0 (0.0) | 134 (5.4) |  |
| 25.0-29.9 | 1 (100.0) | 1003 (32.7) |  | 0 (0.0) | 605 (24.6) |  |
| 30.0-34.9 | 0 (0.0) | 1339 (43.7) |  | 0 (0.0) | 1035 (42.0) |  |
| 35.0-39.9 | 0 (0.0) | 404 (13.2) |  | 0 (0.0) | 479 (19.4) |  |
| ≥40.0 | 0 (0.0) | 44 (1.4) |  | 0 (0.0) | 211 (8.6) |  |
| *Missing* | *262 (99.6)* | *502 (14.1)* |  | *147 (100.0)* | *332 (11.9)* |  |
| Age at conception, median (IQR), y | 29.2 [25.4, 33.0] | 31.5 [28.8, 34.3] | *<0.01* | 32.0 [27.8, 36.2] | 33.2 [30.2, 36.5] | *<0.01* |
| <25.0 | 64 (24.4) | 232 (7.0) |  | 21 (14.3) | 111 (4.0) |  |
| 25.0-29.9 | 84 (32.1) | 918 (27.7) |  | 40 (27.2) | 556 (20.2) |  |
| 30.0-34.9 | 71 (27.1) | 1469 (44.4) |  | 41 (27.9) | 1097 (39.8) |  |
| 35.0-39.9 | 38 (14.5) | 597 (18.0) |  | 34 (23.1) | 658 (23.9) |  |
| ≥40.0 | 5 (1.9) | 94 (2.8) |  | 11 (7.5) | 334 (12.1) |  |
| *Missing* | *1 (0.4)* | *259 (7.3)* |  | *0 (0.0)* | *40 (1.4)* |  |
| Ethnicity |  |  | *<0.01* |  |  | *<0.01* |
| Dutch | 31 (30.7) | 2025 (60.6) |  | 26 (40.6) | 1687 (64.3) |  |
| European^c^ | 11 (10.9) | 343 (10.3) |  | 5 (7.8) | 221 (8.4) |  |
| Non-European^d^ | 59 (58.4) | 975 (29.2) |  | 33 (51.6) | 716 (27.3) |  |
| *Missing* | *162 (61.6)* | *226 (6.3)* |  | *83 (56.5)* | *172 (6.2)* |  |
| Educational level |  |  | *<0.01* |  |  |  |
| No, primary, or secondary education finished | 52 (52.5) | 994 (29.9) |  | 33 (52.4) | 949 (35.9) |  |
| Higher education finished | 47 (47.5) | 2329 (70.1) |  | 30 (47.6) | 1691 (64.1) | *0.01* |
| *Missing* | *164 (62.4)* | *246 (6.9)* |  | *84 (57.1)* | *156 (5.6)* |  |
| BMI, median (IQR), kg/m^2^ | 24.2 [21.5, 28.1] | 23.6 [21.3, 26.9] | *0.41* | 25.7 [23.0, 28.7] | 25.0 [23.0, 27.5] | *0.04* |
| *Missing* | *163 (62.0)* | *96 (2.7)* |  | *127 (86.4)* | *268 (9.6)* |  |
| Smoking |  |  | *<0.01* |  |  | *0.50* |
| No | 14 (30.4) | 1615 (54.7) |  | 8 (40.0) | 1262 (51.2) |  |
| No, quit smoking before pregnancy | 7 (15.2) | 919 (31.1) |  | 5 (25.0) | 598 (24.3) |  |
| Yes, smoked during pregnancy | 25 (54.3) | 421 (14.2) |  | 7 (35.0) | 603 (24.5) |  |
| *Missing* | *217 (82.5)* | *614 (17.2)* |  | *127 (86.4)* | *333 (11.9)* |  |
| Alcohol consumption |  |  | *<0.01* |  |  | *0.25* |
| No consumption < 3 months before pregnancy | 29 (46.8) | 695 (22.1) |  | 5 (22.7) | 307 (12.3) |  |
| Consumption < 3 months before pregnancy | 20 (32.3) | 1983 (63.1) |  | 17 (77.3) | 2179 (87.7) |  |
| Consumption during pregnancy | 13 (21.0) | 463 (14.7) |  | NA | NA |  |
| *Missing* | *201 (76.4)* | *428 (12.0)* |  | *125 (85.0)* | *310 (11.1)* |  |
| Parity |  |  | *<0.01* |  |  | *<0.01* |
| Nulliparous | 21 (34.4) | 2069 (64.6) |  | 16 (39.0)^f^ | 1686 (65.3) ^f^ |  |
| Multiparous | 40 (65.6) | 2446 (77.9) |  | 25 (61.0) ^f^ | 895 (34.7) ^f^ |  |
| *Missing* | *202 (76.8)* | *365 (10.2)* |  | *106 (72.1) ^f^* | *215 (7.7) ^f^* |  |
| Miscarriage in previous pregnancy |  |  | *0.75* |  |  | *0.26* |
| No | 25 (83.3) | 2312 (79.3) |  | 16 (94.1) ^f^ | 1867 (80.3) ^f^ |  |
| Yes | 5 (16.7) | 603 (20.7) |  | 1 (5.9) ^f^ | 459 (19.7) ^f^ |  |
| *Missing* | *233 (88.6)* | *654 (18.3)* |  | *130 (88.4) ^f^* | *470 (16.8) ^f^* |  |
| Time to pregnancy in months, median (95% range), mo.^e^ | NA | 3.8 [0.0-68.2] | *NA* | NA ^f^ | 3.5 [0.0-60.4] ^f^ | *NA* |
| ≤12 months | 0 (0.0) | 1964 (63.6) |  | 0 (0.0) ^f^ | 1788 (72.2) ^f^ |  |
| >12 months | 0 (0.0) | 556 (18.0) |  | 0 (0.0) ^f^ | 434 (17.5) ^f^ |  |
| ART leading to pregnancy | 9 (90.0) | 310 (10.0) |  | 3 (100.0) ^f^ | 241 (9.7) ^f^ |  |
| Not pregnant | 1 (10.0) | 259 (8.4) |  | 0 (0.0) ^f^ | 13 (0.5) ^f^ |  |
| *Missing* | *253 (96.2)* | *480 (13.4)* |  | *144 (100.0) ^f^* | *320 (11.4) ^f^* |  |
| Occurrence of miscarriage |  |  |  |  |  | *NA* |
| No miscarriage | 262 (100.0) | 2951 (89.2) |  | 147 (100.0) ^f^ | 2530 (90.9) ^f^ |  |
| Miscarriage | 0 (0.0) | 359 (10.8) |  | 0 (0.0) ^f^ | 253 (9.1) ^f^ |  |
| *Missing* | *1 (0.4)* | *259 (7.3)* |  | *0 (0.0) ^f^* | *13 (0.5) ^f^* |  |
| Timing of miscarriage, median (IQR), wk. | NA | 8.1 [7.0, 9.4] | *NA* | NA ^f^ | 8.3 [7.1, 9.4] ^f^ | *NA* |
| First trimester | 0 (0.0) | 328 (93.2) |  | 0 (0.0) ^f^ | 234 (94.0) ^f^ |  |
| Second trimester | 0 (0.0) | 25 (6.8) |  | 0 (0.0) ^f^ | 15 (6.0) ^f^ |  |
| *Missing* | *263 (100.0)* | *7 (1.9)* |  | *147 (100.0) ^f^* | *4 (1.6) ^f^* |  |
| Abbreviations: ART, assisted reproductive technology; BMI, body mass index (calculated as weight in kilograms divided by height in meters squared); NA, not applicable.  Women were included in preconception and pregnancy between 2017 and 2021. Values are presented as median (IQR), median (95% range) or number of participants (valid %).   1. Study population of time to pregnancy and miscarriage consisting of 3,224 unique women and 2,511 unique men from Rotterdam, the Netherlands with a total of 3,569 and 2,796 study episodes. 2. Non-responders consisted of 262 unique women and 147 unique men from Rotterdam, the Netherlands with a total of 263 and 147 study episodes. 3. Included: European, German, Yugoslav, or Polish ethnicity. 4. Included: African; American, non-western; Asian, non-western; Chinese; Indonesian; American, western; Asian, western; Cape Verdean; Dutch Antilles; Moroccan; Oceanian; Surinamese, or Turkish ethnicity. 5. Time to pregnancy in months was derived from pregnancy episodes with a natural conception. 6. Parity, miscarriage in previous pregnancy, time to pregnancy in months, occurrence of miscarriage, timing of miscarriage in weeks in men were derived from their partner. | | | | | | |

## Table S3. Population Characteristics stratified per Group of Age at Pursuing Pregnancy of Female Episodes.

|  | **Women** | | | | | |
| --- | --- | --- | --- | --- | --- | --- |
|  | **Episodes, No. (%)** | | | | | |
|  | Overall | <25.0 years | 25.0-29.9 years | 30.0-34.9 years | 35.0-39.9 years | ≥40 years |
|  | N=3,067 | N=277 | N=1,003 | N=1,339 | N=404 | N=44 |
| Age at pursuing pregnancy, median (IQR), y | 30.8 [28.2, 33.3] | 23.0 [21.2, 24.3] | 28.3 [27.1, 29.3] | 32.0 [31.0, 33.2] | 36.6 [35.7, 37.9] | 41.2 [40.5, 42.0] |
| Ethnicity |  |  |  |  |  |  |
| Dutch | 1887 (61.9) | 142 (51.6) | 662 (66.3) | 859 (64.5) | 203 (50.6) | 21 (48.8) |
| European^a^ | 313 (10.3) | 19 (6.9) | 84 (8.4) | 151 (11.3) | 56 (14.0) | 3 (7.0) |
| Non-European^b^ | 849 (27.8) | 114 (41.5) | 252 (25.3) | 322 (24.2) | 142 (35.4) | 19 (44.2) |
| *Missing* | *18 (0.6)* | *2 (0.7)* | *5 (0.5)* | *7 (0.5)* | *3 (0.7)* | *1 (2.3)* |
| Educational level |  |  |  |  |  |  |
| No, primary, or secondary education finished | 866 (28.6) | 214 (78.4) | 327 (32.9) | 222 (16.8) | 87 (22.0) | 16 (36.4) |
| Higher education finished | 2165 (71.4) | 59 (21.6) | 666 (67.1) | 1103 (83.2) | 309 (78.0) | 28 (63.6) |
| *Missing* | *36 (1.2)* | *4 (1.4)* | *10 (1.0)* | *14 (1.0)* | *8 (2.0)* | *0* |
| BMI, median (IQR), kg/m^2^ | 23.5 [21.2, 26.5] | 25.4 [22.6, 29.6] | 23.3 [21.2, 26.5] | 23.0 [21.0, 25.7] | 23.9 [21.5, 27.4] | 25.5 [23.3, 29.5] |
| *Missing* | *39 (1.3)* | *9 (3.2)* | *12 (1.2)* | *10 (0.7)* | *7 (1.7)* | *1 (2.3)* |
| Smoking |  |  |  |  |  |  |
| No | 1569 (55.2) | 109 (43.4) | 533 (56.2) | 727 (59.1) | 179 (48.4) | 21 (53.8) |
| No, quit smoking before pregnancy | 891 (31.4) | 64 (25.5) | 292 (30.8) | 371 (30.1) | 151 (40.8) | 13 (33.3) |
| Yes, smoked during pregnancy | 380 (13.4) | 78 (31.1) | 124 (13.1) | 133 (10.8) | 40 (10.8) | 5 (12.8) |
| *Missing* | *227 (7.4)* | *26 (9.4)* | *54 (5.4)* | *108 (8.1)* | *34 (8.4)* | *5 (11.4)* |
| Alcohol consumption |  |  |  |  |  |  |
| No consumption < 3 months before pregnancy | 626 (21.1) | 95 (37.1) | 227 (23.4) | 198 (15.1) | 94 (24.1) | 12 (31.6) |
| Consumption < 3 months before pregnancy | 1912 (64.4) | 128 (50.0) | 609 (62.8) | 915 (69.6) | 238 (61.0) | 22 (57.9) |
| Consumption during pregnancy | 431 (14.5) | 33 (12.9) | 134 (13.8) | 202 (15.4) | 58 (14.9) | 4 (10.5) |
| *Missing* | *98 (3.2)* | *21 (7.6)* | *33 (3.3)* | *24 (1.8)* | *14 (3.5)* | *6 (13.6)* |
| Parity |  |  |  |  |  |  |
| Nulliparous | 1952 (65.6) | 180 (71.1) | 715 (73.5) | 845 (64.2) | 195 (49.9) | 17 (41.5) |
| Multiparous | 1022 (34.4) | 73 (28.9) | 258 (26.5) | 471 (35.8) | 196 (50.1) | 24 (58.5) |
| *Missing* | *93 (3.0)* | *24 (8.7)* | *30 (3.0)* | *23 (1.7)* | *13 (3.2)* | *3 (6.8)* |
| Miscarriage in previous pregnancy |  |  |  |  |  |  |
| No | 2235 (79.3) | 184 (75.4) | 771 (81.8) | 1006 (81.9) | 256 (69.8) | 18 (46.2) |
| Yes | 585 (20.7) | 60 (24.6) | 171 (18.2) | 222 (18.1) | 111 (30.2) | 21 (53.8) |
| *Missing* | *247 (8.1)* | *33 (11.9)* | *61 (6.1)* | *111 (8.3)* | *37 (9.2)* | *5 (11.4)* |
| Time to pregnancy in months, median (95% range), mo.^c^ | 3.8 [0.0, 67.9] | 8.0 [0.0, 147.6] | 3.5 [0.0, 68.4] | 3.3 [0.0, 52.7] | 4.0 [0.0, 40.5] | 5.0 [0.0, 38.8] |
| ≤12 months | 1964 (64.0) | 132 (47.7) | 639 (63.7) | 920 (68.7) | 252 (62.4) | 21 (47.7) |
| >12 months | 554 (18.1) | 89 (32.1) | 197 (19.6) | 201 (15.0) | 61 (15.1) | 6 (13.6) |
| ART leading to pregnancy | 290 (9.5) | 28 (10.1) | 87 (8.7) | 126 (9.4) | 45 (11.1) | 4 (9.1) |
| Not pregnant | 259 (8.4) | 28 (10.1) | 80 (8.0) | 92 (6.9) | 46 (11.4) | 13 (29.5) |
| *Missing* | *0* | *0* | *0* | *0* | *0* | *0* |
| Occurrence of miscarriage |  |  |  |  |  |  |
| No miscarriage | 2587 (92.1) | 236 (94.8) | 857 (92.8) | 1159 (92.9) | 311 (86.9) | 24 (77.4) |
| Miscarriage | 221 (7.9) | 13 (5.2) | 66 (7.2) | 88 (7.1) | 47 (13.1) | 7 (22.6) |
| *Missing* | *259 (8.4)* | *28 (10.1)* | *80 (8.0)* | *92 (6.9)* | *46 (11.4)* | *13 (29.5)* |
| Timing of miscarriage, median (IQR), wk. | 8.1 [7.0, 9.4] | 8.6 [7.4, 8.7] | 8.1 [7.1, 9.6] | 7.9 [6.9, 8.9] | 8.6 [7.2, 10.6] | 9.3 [7.9, 10.3] |
| First trimester | 203 (92.7) | 11 (84.6) | 59 (95.2) | 85 (96.6) | 41 (87.2) | 7 (100.0) |
| Second trimester | 16 (7.3) | 2 (15.4) | 5 (4.8) | 3 (3.4) | 6 (12.8) | 0 (0.0) |
| *Missing* | *3 (1.4)* | *0 (0.0)* | *3 (4.5)* | *0 (0.0)* | *0 (0.0)* | *0 (0.0)* |
| Abbreviations: ART, assisted reproductive technology; BMI, body mass index (calculated as weight in kilograms divided by height in meters squared); NA, not applicable.  Women were included in preconception and pregnancy between 2017 and 2021. Values are presented as median (IQR), median (95% range) or number of participants (valid %).   1. Included: European, German, Yugoslav, or Polish. 2. Included: African; American, non-western; Asian, non-western; Chinese; Indonesian; American, western; Asian, western; Cape Verdean; Dutch Antilles; Moroccan; Oceanian; Surinamese, or Turkish ethnicity. 3. Time to pregnancy in months was derived from pregnancy episodes with a natural conception. | | | | | | |

## Table S4. Population Characteristics stratified per Group of Age at Pursuing Pregnancy of Male Episodes.

|  | **Men** | | | | | |
| --- | --- | --- | --- | --- | --- | --- |
|  | **Episodes, No. (%)** | | | | | |
|  | Overall | <25.0 years | 25.0-29.9 years | 30.0-34.9 years | 35.0-39.9 years | ≥40 years |
|  | N=2,464 | N=134 | N=605 | N=1,035 | N=479 | N=211 |
| Age at pursuing pregnancy, median (IQR), y | 32.1 [29.4, 35.4] | 23.7 [22.1, 24.3] | 28.3 [27.1, 29.2] | 32.2 [31.2, 33.6] | 36.6 [35.8, 38.0] | 42.5 [41.2, 45.2] |
| Ethnicity |  |  |  |  |  |  |
| Dutch | 1586 (65.3) | 72 (54.5) | 379 (63.9) | 700 (68.5) | 308 (64.4) | 127 (62.3) |
| European^a^ | 202 (8.3) | 5 (3.8) | 39 (6.6) | 90 (8.8) | 54 (11.3) | 14 (6.9) |
| Non-European^b^ | 641 (26.4) | 55 (41.7) | 175 (29.5) | 232 (22.7) | 116 (24.3) | 63 (30.9) |
| *Missing* | *35 (1.4)* | *2 (1.5)* | *12 (2.0)* | *13 (1.3)* | *1 (0.2)* | *7 (3.3)* |
| Educational level |  |  |  |  |  |  |
| No, primary, or secondary education finished | 857 (35.0) | 101 (77.1) | 258 (42.9) | 281 (27.3) | 131 (27.5) | 86 (41.5) |
| Higher education finished | 1589 (65.0) | 30 (22.9) | 344 (57.1) | 748 (72.7) | 346 (72.5) | 121 (58.5) |
| *Missing* | *18 (0.7)* | *3 (2.2)* | *3 (0.5)* | *6 (0.6)* | *2 (0.4)* | *4 (1.9)* |
| BMI, median (IQR), kg/m^2^ | 24.9 [23.0, 27.4] | 25.6 [23.1, 29.2] | 25.1 [22.9, 27.4] | 24.5 [22.8, 26.8] | 25.3 [23.0, 27.6] | 25.6 [24.1, 27.9] |
| *Missing* | *166 (6.7)* | *11 (8.2)* | *38 (6.3)* | *68 (6.6)* | *30 (6.3)* | *19 (9.0)* |
| Smoking |  |  |  |  |  |  |
| No | 1227 (51.5) | 50 (40.3) | 324 (55.2) | 553 (55.0) | 212 (45.1) | 88 (44.7) |
| No, quit smoking before pregnancy | 582 (24.4) | 23 (18.5) | 101 (17.2) | 236 (23.5) | 156 (33.2) | 66 (33.5) |
| Yes, smoked during pregnancy | 575 (24.1) | 51 (41.1) | 162 (27.6) | 217 (21.6) | 102 (21.7) | 43 (21.8) |
| *Missing* | *80 (3.2)* | *10 (7.5)* | *18 (3.0)* | *29 (2.8)* | *9 (1.9)* | *14 (6.6)* |
| Alcohol consumption |  |  |  |  |  |  |
| No consumption < 3 months before pregnancy | 291 (12.1) | 27 (21.6) | 87 (14.7) | 95 (9.3) | 51 (10.9) | 31 (15.3) |
| Consumption < 3 months before pregnancy | 2113 (87.9) | 98 (78.4) | 504 (85.3) | 922 (90.7) | 418 (89.1) | 171 (84.7) |
| Consumption during pregnancy | NA | NA | *NA* | NA | NA | *NA* |
| *Missing* | *60 (2.4)* | *9 (6.7)* | *14 (2.3)* | *18 (1.7)* | *10 (2.1)* | *9 (4.3)* |
| Parity^c^ |  |  |  |  |  |  |
| Nulliparous | 1601 (66.3) | 88 (68.8) | 423 (71.9) | 705 (69.0) | 265 (56.6) | 120 (58.0) |
| Multiparous | 812 (33.7) | 40 (31.2) | 165 (28.1) | 317 (31.0) | 203 (43.4) | 87 (42.0) |
| *Missing* | *51 (2.1)* | *6 (4.5)* | *17 (2.8)* | *13 (1.3)* | *11 (2.3)* | *4 (1.9)* |
| Miscarriage in previous pregnancy ^c^ |  |  |  |  |  |  |
| No | 1809 (80.2) | 92 (74.8) | 456 (81.9) | 788 (82.4) | 340 (78.2) | 133 (71.9) |
| Yes | 447 (19.8) | 31 (25.2) | 101 (18.1) | 168 (17.6) | 95 (21.8) | 52 (28.1) |
| *Missing* | *208 (8.4)* | *11 (8.2)* | *48 (7.9)* | *79 (7.6)* | *44 (9.2)* | *26 (12.3)* |
| Time to pregnancy in months, median (95% range), mo.^c, d^ | 3.5 [0.0, 59.9] | 6.2 [0.0, 104.7] | 3.3 [0.0, 60.4] | 3.0 [0.0, 53.9] | 3.6 [0.0, 52.0] | 4.3 [0.0, 48.1] |
| ≤12 months | 1788 (72.6) | 83 (61.9) | 443 (73.2) | 762 (73.6) | 350 (73.1) | 150 (71.1) |
| >12 months | 433 (17.6) | 39 (29.1) | 103 (17.0) | 179 (17.3) | 80 (16.7) | 32 (15.2) |
| ART leading to pregnancy | 230 (9.3) | 12 (9.0) | 55 (9.1) | 88 (8.5) | 48 (10.0) | 27 (12.8) |
| Not pregnant | 13 (0.5) | 0 (0.0) | 4 (0.7) | 6 (0.6) | 1 (0.2) | 2 (0.9) |
| *Missing* | *0* | *0* | *0* | *0* | *0* | *0* |
| Occurrence of miscarriage ^c^ |  |  |  |  |  |  |
| No miscarriage | 2283 (93.1) | 127 (94.8) | 568 (94.5) | 962 (93.5) | 444 (92.9) | 182 (87.1) |
| Miscarriage | 168 (6.9) | 7 (5.2) | 33 (5.5) | 67 (6.5) | 34 (7.1) | 27 (12.9) |
| *Missing* | *13 (0.5)* | *0 (0.0)* | *4 (0.7)* | *6 (0.6)* | *1 (0.2)* | *2 (0.9)* |
| Timing of miscarriage, median (IQR), wk. ^c^ | 8.4 [7.1, 9.4] | 8.6 [7.9, 13.6] | 8.6 [7.4, 10.0] | 8.0 [6.9, 9.1] | 8.1 [7.5, 9.4] | 8.4 [7.8, 9.2] |
| First trimester | 155 (92.8) | 5 (71.4) | 29 (87.9) | 63 (94.0) | 32 (94.1) | 26 (100.0) |
| Second trimester | 12 (7.2) | 2 (28.6) | 4 (12.1) | 4 (6.0) | 2 (5.9) | 0 (0.0) |
| *Missing* | *1 (0.6)* | *0 (0.0)* | *0 (0.0)* | *0 (0.0)* | *0 (0.0)* | *1 (3.7)* |
| Abbreviations: ART, assisted reproductive technology; BMI, body mass index (calculated as weight in kilograms divided by height in meters squared); NA, not applicable.  Women were included in preconception and pregnancy between 2017 and 2021. Values are presented as median (IQR), median (95% range) or number of participants (valid %).   1. Included: European, German, Yugoslav, or Polish. 2. Included: African; American, non-western; Asian, non-western; Chinese; Indonesian; American, western; Asian, western; Cape Verdean; Dutch Antilles; Moroccan; Oceanian; Surinamese, or Turkish ethnicity. 3. Parity, miscarriage in previous pregnancy, time to pregnancy in months, occurrence of miscarriage, timing of miscarriage in weeks in men were derived from their partner. 4. Time to pregnancy in months was derived from pregnancy episodes with a natural conception. | | | | | | |

## Table S5. Population Characteristics stratified per Group of Age at Conception of Female Episodes.

|  | **Women** | | | | | |
| --- | --- | --- | --- | --- | --- | --- |
|  | **Episodes, No. (%)** | | | | | |
|  | Overall | <25.0 years | 25.0-29.9 years | 30.0-34.9 years | 35.0-39.9 years | ≥40 years |
|  | N=2,831 | N=186 | N=771 | N=1,289 | N=508 | N=77 |
| Age at conception, median (IQR), y | 31.5 [28.9, 34.3] | 22.9 [21.2, 24.3] | 28.2 [26.9, 29.2] | 32.1 [31.0, 33.4] | 36.7 [35.9, 38.0] | 41.1 [40.6, 42.0] |
| Ethnicity |  |  |  |  |  |  |
| Dutch | 1647 (63.1) | 70 (48.3) | 450 (64.5) | 827 (67.5) | 267 (56.6) | 33 (46.5) |
| European^a^ | 256 (9.8) | 11 (7.6) | 68 (9.7) | 109 (8.9) | 57 (12.1) | 11 (15.5) |
| Non-European^b^ | 708 (27.1) | 64 (44.1) | 180 (25.8) | 289 (23.6) | 148 (31.4) | 27 (38.0) |
| *Missing* | *220 (7.8)* | *41 (22.0)* | *73 (9.5)* | *64 (5.0)* | *36 (7.1)* | *6 (7.8)* |
| Educational level |  |  |  |  |  |  |
| No, primary, or secondary education finished | 736 (28.4) | 124 (86.1) | 266 (38.5) | 223 (18.3) | 105 (22.3) | 18 (25.4) |
| Higher education finished | 1856 (71.6) | 20 (13.9) | 425 (61.5) | 993 (81.7) | 365 (77.7) | 53 (74.6) |
| *Missing* | *239 (8.4)* | *42 (22.6)* | *73 (9.5)* | *73 (5.7)* | *38 (7.5)* | *6 (7.8)* |
| BMI, median (IQR), kg/m^2^ | 23.5 [21.3, 26.7] | 25.2 [21.9, 29.3] | 23.6 [21.4, 27.1] | 23.2 [21.2, 26.1] | 23.8 [21.3, 26.7] | 25.5 [23.4, 29.3] |
| *Missing* | *62 (2.2)* | *3 (1.6)* | *14 (1.8)* | *27 (2.1)* | *14 (2.8)* | *4 (5.2)* |
| Smoking |  |  |  |  |  |  |
| No | 1251 (55.2) | 47 (39.5) | 351 (55.6) | 644 (60.1) | 185 (46.7) | 24 (48.0) |
| No, quit smoking before pregnancy | 682 (30.1) | 28 (23.5) | 175 (27.7) | 305 (28.5) | 155 (39.1) | 19 (38.0) |
| Yes, smoked during pregnancy | 335 (14.8) | 44 (37.0) | 105 (16.6) | 123 (11.5) | 56 (14.1) | 7 (14.0) |
| *Missing* | *563 (19.9)* | *67 (36.0)* | *140 (18.2)* | *217 (16.8)* | *112 (22.0)* | *27 (35.1)* |
| Alcohol consumption |  |  |  |  |  |  |
| No consumption < 3 months before pregnancy | 529 (21.6) | 43 (34.4) | 171 (26.1) | 198 (17.0) | 101 (22.5) | 16 (29.1) |
| Consumption < 3 months before pregnancy | 1507 (61.5) | 66 (52.8) | 377 (57.6) | 767 (65.7) | 262 (58.5) | 35 (63.6) |
| Consumption during pregnancy | 413 (16.9) | 16 (12.8) | 106 (16.2) | 202 (17.3) | 85 (19.0) | 4 (7.3) |
| *Missing* | *382 (13.5)* | *61 (32.8)* | *117 (15.2)* | *122 (9.5)* | *60 (11.8)* | *22 (28.6)* |
| Parity |  |  |  |  |  |  |
| Nulliparous | 1596 (63.7) | 89 (76.1) | 503 (75.6) | 757 (63.2) | 220 (47.8) | 27 (39.7) |
| Multiparous | 911 (36.3) | 28 (23.9) | 162 (24.4) | 440 (36.8) | 240 (52.2) | 41 (60.3) |
| *Missing* | *324 (11.4)* | *69 (37.1)* | *106 (13.7)* | *92 (7.1)* | *48 (9.4)* | *9 (11.7)* |
| Miscarriage in previous pregnancy |  |  |  |  |  |  |
| No | 1783 (79.8) | 89 (80.2) | 527 (84.7) | 870 (82.2) | 267 (68.1) | 30 (60.0) |
| Yes | 451 (20.2) | 22 (19.8) | 95 (15.3) | 189 (17.8) | 125 (31.9) | 20 (40.0) |
| *Missing* | *597 (21.1)* | *75 (40.3)* | *149 (19.3)* | *230 (17.8)* | *116 (22.8)* | *27 (35.1)* |
| Time to pregnancy in months, median (95% range), mo.^c^ | 3.6 [0.0, 65.3] | 3.0 [0.0, 40.0] | 2.6 [0.0, 49.6] | 3.5 [0.0, 53.7] | 6.4 [0.0, 106.2] | 10.6 [0.0, 157.4] |
| ≤12 months | 1650 (70.2) | 83 (79.0) | 483 (76.2) | 828 (73.7) | 234 (54.5) | 22 (36.7) |
| >12 months | 436 (18.5) | 19 (18.1) | 104 (16.4) | 181 (16.1) | 113 (26.3) | 19 (31.7) |
| ART leading to pregnancy | 265 (11.3) | 3 (2.9) | 47 (7.4) | 114 (10.2) | 82 (19.1) | 19 (31.7) |
| Not pregnant | NA | NA | NA | NA | NA | NA |
| *Missing* | *480 (17.0)* | *81 (43.5)* | *137 (17.8)* | *166 (12.9)* | *79 (15.6)* | *17 (22.1)* |
| Occurrence of miscarriage |  |  |  |  |  |  |
| No miscarriage | 2472 (87.3) | 174 (93.5) | 687 (89.1) | 1152 (89.4) | 409 (80.5) | 50 (64.9) |
| Miscarriage | 359 (12.7) | 12 (6.5) | 84 (10.9) | 137 (10.6) | 99 (19.5) | 27 (35.1) |
| *Missing* | *0* | *0* | *0* | *0* | *0* | *0* |
| Timing of miscarriage, median (IQR), wk. | 8.1 [7.0, 9.4] | 8.6 [7.0, 12.5] | 8.3 [7.3, 9.6] | 8.0 [6.9, 9.3] | 8.1 [7.0, 9.1] | 8.6 [7.0, 9.1] |
| First trimester | 328 (92.9) | 8 (72.7) | 73 (89.0) | 132 (97.8) | 90 (91.8) | 25 (92.6) |
| Second trimester | 25 (7.1) | 3 (27.3) | 9 (11.0) | 3 (2.2) | 8 (8.2) | 2 (7.4) |
| *Missing* | *7 (1.9)* | *1 (8.3)* | *3 (3.5)* | *2 (1.5)* | *1 (1.0)* | *0 (0.0)* |
| Abbreviations: ART, assisted reproductive technology; BMI, body mass index (calculated as weight in kilograms divided by height in meters squared); NA, not applicable.  Women were included in preconception and pregnancy between 2017 and 2021. Values are presented as median (IQR), median (95% range) or number of participants (valid %).   1. Included: European, German, Yugoslav, or Polish. 2. Included: African; American, non-western; Asian, non-western; Chinese; Indonesian; American, western; Asian, western; Cape Verdean; Dutch Antilles; Moroccan; Oceanian; Surinamese, or Turkish ethnicity. 3. Time to pregnancy in months was derived from pregnancy episodes with a natural conception. | | | | | | |

## Table S6. Population Characteristics stratified per Group of Age at Conception of Male Episodes.

|  | **Men** | | | | | |
| --- | --- | --- | --- | --- | --- | --- |
|  | **Episodes, No. (%)** | | | | | |
|  | Overall | <25.0 years | 25.0-29.9 years | 30.0-34.9 years | 35.0-39.9 years | ≥40 years |
|  | N=2,392 | N=98 | N=483 | N=947 | N=579 | N=285 |
| Age at conception, median (IQR), y | 33.3 [30.2, 36.5] | 23.4 [22.1, 24.4] | 28.4 [27.1, 29.3] | 32.6 [31.4, 33.8] | 36.7 [35.8, 38.0] | 42.7 [41.2, 45.7] |
| Ethnicity |  |  |  |  |  |  |
| Dutch | 1455 (65.2) | 43 (54.4) | 274 (62.8) | 611 (68.1) | 349 (63.7) | 178 (65.2) |
| European^a^ | 180 (8.1) | 0 (0.0) | 26 (6.0) | 72 (8.0) | 64 (11.7) | 18 (6.6) |
| Non-European^b^ | 598 (26.8) | 36 (45.6) | 136 (31.2) | 214 (23.9) | 135 (24.6) | 77 (28.2) |
| *Missing* | *159 (6.6)* | *19 (19.4)* | *47 (9.7)* | *50 (5.3)* | *31 (5.4)* | *12 (4.2)* |
| Educational level |  |  |  |  |  |  |
| No, primary, or secondary education finished | 783 (34.9) | 65 (81.2) | 207 (46.8) | 259 (28.8) | 150 (27.5) | 102 (37.1) |
| Higher education finished | 1459 (65.1) | 15 (18.8) | 235 (53.2) | 640 (71.2) | 396 (72.5) | 173 (62.9) |
| *Missing* | *150 (6.3)* | *18 (18.4)* | *41 (8.5)* | *48 (5.1)* | *33 (5.7)* | *10 (3.5)* |
| BMI, median (IQR), kg/m^2^ | 25.0 [23.0, 27.5] | 25.1 [22.3, 28.7] | 25.1 [23.0, 27.7] | 24.5 [22.7, 26.6] | 25.4 [23.3, 28.0] | 25.6 [23.9, 28.0] |
| *Missing* | *200 (8.4)* | *15 (15.3)* | *38 (7.9)* | *72 (7.6)* | *50 (8.6)* | *25 (8.8)* |
| Smoking |  |  |  |  |  |  |
| No | 1059 (50.9) | 25 (36.2) | 222 (53.4) | 472 (55.9) | 236 (46.6) | 104 (42.4) |
| No, quit smoking before pregnancy | 519 (24.9) | 15 (21.7) | 74 (17.8) | 177 (20.9) | 164 (32.4) | 89 (36.3) |
| Yes, smoked during pregnancy | 503 (24.2) | 29 (42.0) | 120 (28.8) | 196 (23.2) | 106 (20.9) | 52 (21.2) |
| *Missing* | *311 (13.0)* | *29 (29.6)* | *67 (13.9)* | *102 (10.8)* | *73 (12.6)* | *40 (14.0)* |
| Alcohol consumption |  |  |  |  |  |  |
| No consumption < 3 months before pregnancy | 247 (11.8) | 11 (15.9) | 64 (15.2) | 77 (9.0) | 62 (12.3) | 33 (13.2) |
| Consumption < 3 months before pregnancy | 1854 (88.2) | 58 (84.1) | 356 (84.8) | 779 (91.0) | 444 (87.7) | 217 (86.8) |
| Consumption during pregnancy | NA | NA | NA | NA | NA | NA |
| *Missing* | *291 (12.2)* | *29 (29.6)* | *63 (13.0)* | *91 (9.6)* | *73 (12.6)* | *35 (12.3)* |
| Parity ^c^ |  |  |  |  |  |  |
| Nulliparous | 1440 (65.7) | 56 (80.0) | 328 (77.0) | 617 (69.6) | 305 (56.6) | 134 (49.6) |
| Multiparous | 751 (34.3) | 14 (20.0) | 98 (23.0) | 269 (30.4) | 234 (43.4) | 136 (50.4) |
| *Missing* | *201 (8.4)* | *28 (28.6)* | *57 (11.8)* | *61 (6.4)* | *40 (6.9)* | *15 (5.3)* |
| Miscarriage in previous pregnancy ^c^ |  |  |  |  |  |  |
| No | 1581 (81.0) | 53 (77.9) | 340 (85.4) | 674 (84.7) | 350 (74.6) | 164 (73.9) |
| Yes | 372 (19.0) | 15 (22.1) | 58 (14.6) | 122 (15.3) | 119 (25.4) | 58 (26.1) |
| *Missing* | *439 (18.4)* | *30 (30.6)* | *85 (17.6)* | *151 (15.9)* | *110 (19.0)* | *63 (22.1)* |
| Time to pregnancy in months, median (95% range), mo.^c, d^ | 3.5 [0.0, 58.1] | 2.9 [0.0, 58.1] | 3.0 [0.0, 49.3] | 2.9 [0.0, 54.3] | 4.4 [0.0, 70.8] | 6.2 [0.0, 54.8] |
| ≤12 months | 1519 (73.3) | 54 (84.4) | 336 (81.2) | 656 (77.5) | 329 (65.5) | 144 (58.5) |
| >12 months | 349 (16.8) | 9 (14.1) | 53 (12.8) | 130 (15.4) | 104 (20.7) | 53 (21.5) |
| ART leading to pregnancy | 204 (9.8) | 1 (1.6) | 25 (6.0) | 60 (7.1) | 69 (13.7) | 49 (19.9) |
| Not pregnant | NA | NA | NA | NA | NA | NA |
| *Missing* | *320 (13.4)* | *34 (34.7)* | *69 (14.3)* | *101 (10.7)* | *77 (13.3)* | *39 (13.7)* |
| Occurrence of miscarriage ^c^ |  |  |  |  |  |  |
| No miscarriage | 2142 (89.5) | 88 (89.8) | 441 (91.3) | 858 (90.6) | 520 (89.8) | 235 (82.5) |
| Miscarriage | 250 (10.5) | 10 (10.2) | 42 (8.7) | 89 (9.4) | 59 (10.2) | 50 (17.5) |
| *Missing* | *0* | *0* | *0* | *0* | *0* | *0* |
| Timing of miscarriage, median (IQR), wk. ^c^ | 8.3 [7.1, 9.4] | 11.6 [8.3, 13.4] | 8.6 [7.4, 9.4] | 8.0 [7.0, 9.1] | 8.1 [6.9, 9.1] | 8.6 [7.9, 9.3] |
| First trimester | 231 (93.9) | 6 (66.7) | 39 (95.1) | 85 (95.5) | 54 (93.1) | 47 (95.9) |
| Second trimester | 15 (6.1) | 3 (33.3) | 2 (4.9) | 4 (4.5) | 4 (6.9) | 2 (4.1) |
| *Missing* | *4 (1.6)* | *1 (10.0)* | *1 (2.4)* | *0 (0.0)* | *1 (1.7)* | *1 (2.0)* |
| Abbreviations: ART, assisted reproductive technology; BMI, body mass index (calculated as weight in kilograms divided by height in meters squared); NA, not applicable.  Women were included in preconception and pregnancy between 2017 and 2021. Values are presented as median (IQR), median (95% range) or number of participants (valid %).   1. Included: European, German, Yugoslav, or Polish. 2. Included: African; American, non-western; Asian, non-western; Chinese; Indonesian; American, western; Asian, western; Cape Verdean; Dutch Antilles; Moroccan; Oceanian; Surinamese, or Turkish ethnicity. 3. Parity, miscarriage in previous pregnancy, time to pregnancy in months, occurrence of miscarriage, timing of miscarriage in weeks in men were derived from their partner. 4. Time to pregnancy in months was derived from pregnancy episodes with a natural conception. | | | | | | |

## Table S7. Population Characteristics stratified per Inclusion Moment.

|  | **Women** | | **Men** | |
| --- | --- | --- | --- | --- |
|  | **Episodes, No. (%)** | | **Episodes, No. (%)** | |
|  | Preconception | Prenatal | Preconception | Prenatal |
| **Characteristic** | N=1,130 | N=2,439 | N=747 | N=2,049 |
| Age at pursuing pregnancy, median (IQR), y | 30.9 [28.8, 33.4] | 30.6 [27.9, 33.3] | 32.4 [29.9, 35.3] | 32.0 [29.2, 35.4] |
| <25.0 | 68 (6.4) | 209 (10.4) | 29 (4.1) | 105 (6.0) |
| 25.0-29.9 | 333 (31.4) | 670 (33.4) | 151 (21.3) | 454 (25.9) |
| 30.0-34.9 | 491 (46.3) | 848 (42.3) | 338 (47.7) | 697 (39.7) |
| 35.0-39.9 | 149 (14.0) | 255 (12.7) | 123 (17.3) | 356 (20.3) |
| ≥40.0 | 20 (1.9) | 24 (1.2) | 68 (9.6) | 143 (8.1) |
| *Missing* | *69 (6.1)* | *433 (17.8)* | *38 (5.1)* | *294 (14.3)* |
| Age at conception, median (IQR), y | 32.0 [29.9, 34.6] | 31.3 [28.4, 34.2] | 33.3 [30.6, 36.4] | 33.2 [29.9, 36.6] |
| <25.0 | 17 (2.0) | 215 (8.8) | 13 (1.8) | 98 (4.8) |
| 25.0-29.9 | 209 (24.0) | 709 (29.1) | 131 (18.2) | 425 (20.9) |
| 30.0-34.9 | 443 (50.9) | 1026 (42.1) | 320 (44.5) | 777 (38.1) |
| 35.0-39.9 | 181 (20.8) | 416 (17.1) | 168 (23.4) | 490 (24.1) |
| ≥40.0 | 21 (2.4) | 73 (3.0) | 87 (12.1) | 247 (12.1) |
| *Missing* | *259 (22.9)* | *0* | *28 (3.7)* | *12 (0.6)* |
| Ethnicity |  |  |  |  |
| Dutch | 677 (61.0) | 1348 (60.4) | 515 (72.4) | 1172 (61.3) |
| European^a^ | 118 (10.6) | 225 (10.1) | 58 (8.2) | 163 (8.5) |
| Non-European^b^ | 315 (28.4) | 660 (29.6) | 138 (19.4) | 578 (30.2) |
| *Missing* | *20 (1.8)* | *206 (8.4)* | *36 (4.8)* | *136 (6.6)* |
| Education level |  |  |  |  |
| No, primary or secondary education finished | 246 (22.3) | 748 (33.7) | 205 (27.9) | 744 (39.1) |
| Higher education finished | 856 (77.7) | 1473 (66.3) | 531 (72.1) | 1160 (60.9) |
| *Missing* | *28 (2.5)* | *218 (8.9)* | *11 (1.5)* | *145 (7.1)* |
| BMI, median (IQR), kg/m^2^ | 23.9 [21.5, 27.0] | 23.5 [21.2, 26.8] | 24.8 [22.9, 27.2] | 25.1 [23.1, 27.6] |
| *Missing* | *0* | *96 (3.9)* | *90 (12.5)* | *178 (8.7)* |
| Smoking |  |  |  |  |
| No | 602 (59.0) | 1013 (52.4) | 378 (55.7) | 884 (49.6) |
| Quit smoking before pregnancy | 374 (36.6) | 545 (28.2) | 188 (27.7) | 410 (23.0) |
| Smoked during pregnancy | 45 (4.4) | 376 (19.4) | 113 (16.6) | 490 (27.5) |
| *Missing* | *109 (9.6)* | *505 (20.7)* | *68 (9.1)* | *265 (12.9)* |
| Alcohol consumption |  |  |  |  |
| No consumption < 3 months before pregnancy | 196 (18.4) | 499 (24.1) | 65 (9.1) | 242 (13.6) |
| Consumption < 3 months before pregnancy | 739 (69.2) | 1244 (60.0) | 647 (90.9) | 1532 (86.4) |
| Consumption during pregnancy | 133 (12.5) | 330 (15.9) | NA | NA |
| *Missing* | *62 (5.5)* | *366 (15.0)* | *35 (4.7)* | *275 (13.4)* |
| Parity |  |  |  |  |
| Nulliparous | 784 (72.7) | 1285 (60.4) | 531 (72.5) ^d^ | 1155 (62.5) ^d^ |
| Multiparous | 294 (27.3) | 841 (39.6) | 201 (27.5) ^d^ | 694 (37.5) ^d^ |
| *Missing* | *52 (4.6)* | *313 (12.8)* | *15 (2.0) ^d^* | *200 (9.8) ^d^* |
| Miscarriage in previous pregnancy |  |  |  |  |
| No | 797 (78.2) | 1515 (79.9) | 538 (79.5) ^d^ | 1329 (80.6) ^d^ |
| Yes | 222 (21.8) | 381 (20.1) | 139 (20.5) ^d^ | 320 (19.4) ^d^ |
| *Missing* | *111 (9.8)* | *543 (22.3)* | *70 (9.4) ^d^* | *400 (19.5) ^d^* |
| Time to pregnancy, median (95% range), mo. ^c^ | 5.7 [0.0, 68.4] | 3.0 [0.0, 67.9] | 5.0 [0.0, 59.4] ^d^ | 2.9 [0.0, 60.4] ^d^ |
| ≤12 months | 496 (46.6) | 1468 (72.5) | 465 (65.4) ^d^ | 1323 (75.0) ^d^ |
| >12 months | 182 (17.1) | 374 (18.5) | 129 (18.1) ^d^ | 305 (17.3) ^d^ |
| ART leading to pregnancy | 127 (11.9) | 183 (9.0) | 104 (14.6) ^d^ | 137 (7.8) ^d^ |
| Not pregnant | 259 (24.3) | 0 (0.0) | 13 (1.8) ^d^ | 0 (0.0) ^d^ |
| *Missing* | *66 (5.8)* | *414 (17.0)* | *36 (4.8) ^d^* | *284 (13.9) ^d^* |
| Occurrence of miscarriage |  |  |  |  |
| No miscarriage | 714 (82.0) | 2237 (91.7) | 631 (86.0) ^d^ | 1899 (92.7) ^d^ |
| Miscarriage | 157 (18.0) | 202 (8.3) | 103 (14.0) ^d^ | 150 (7.3) ^d^ |
| *Missing* | *259 (22.9)* | *0* | *13 (1.7) ^d^* | *0 ^d^* |
| Timing of miscarriage, median (IQR), wk. | 7.9 [6.9, 9.1] | 8.4 [7.3, 9.4] | 8.0 [6.9, 9.1] ^d^ | 8.4 [7.5, 9.4] ^d^ |
| First trimester | 144 (93.5) | 184 (92.9) | 93 (91.2) ^d^ | 141 (95.9) ^d^ |
| Second trimester | 10 (6.5) | 15 (7.1) | 9 (8.8) ^d^ | 6 (3.9) ^d^ |
| *Missing* | *3 (1.9)* | *4 (2.0)* | *1 (1.0) ^d^* | *3 (2.0) ^d^* |
| Abbreviations: ART, assisted reproductive technology; BMI, body mass index (calculated as weight in kilograms divided by height in meters squared); NA, not applicable. Women were included in preconception and pregnancy between 2017 and 2021. Values are presented as median (IQR), median (95% range) or number of participants (valid %).   1. Included: European, German, Yugoslav, or Polish ethnicity. 2. Included: African; American, non-western; Asian, non-western; Chinese; Indonesian; American, western; Asian, western; Cape Verdean; Dutch Antilles; Moroccan; Oceanian; Surinamese, or Turkish ethnicity. 3. Time to pregnancy in months was derived from pregnancy episodes with a natural conception. 4. Parity, miscarriage in previous pregnancy, time to pregnancy in months, occurrence of miscarriage, timing of miscarriage in weeks in men were derived from their partner. | | | | |

# Supplemental Tables of Associations of Age among Women and Men with Fecundability Ratios.

## Table S8. Associations of Age among Women and Men with Fecundability Ratios, Basic Model.

|  | **Women** | | **Men** | |
| --- | --- | --- | --- | --- |
|  | N | FR (95% CI) | N | FR (95% CI) |
| Age at pursuing pregnancy, in years | 2,770 |  | 2,234 |  |
| <25.0 | 249 | **0.56 (0.48-0.65)***** | 122 | **0.64 (0.53-0.78)***** |
| 25.0-29.9 | 913 | **0.89 (0.81-0.97)*** | 550 | 0.95 (0.85-1.06) |
| 30.0-34.9 | 1,210 | Reference | 947 | Reference |
| 35.0-39.9 | 359 | **0.84 (0.74-0.96)**** | 431 | 0.98 (0.87-1.10) |
| ≥40.0 | 39 | **0.52 (0.36-0.74)***** | 184 | 0.95 (0.82-1.09) |
| Values represent the fecundability per categories of age at pursuing pregnancy (95% confidence interval (CI)), as compared to the reference category. Fecundability represents the per-probability of conceiving. Models were analysed using Cox proportional hazards models. Fecundability ratios were derived from the Hazard Ratios of the Cox proportional hazards models.  * p < 0.05, ** p < 0.01, *** p < 0.001 | | | | |

## Table S9. Associations of Age among Women and Men with Fecundability Ratios, Adjusted Model.

|  | **Women** | | **Men** | |
| --- | --- | --- | --- | --- |
|  | N | FR (95% CI) | N | FR (95% CI) |
| Age at pursuing pregnancy, in years | 2,770 |  | 2234 |  |
| <25.0 | 249 | **0.63 (0.54-0.74)***** | 122 | **0.71 (0.58-0.87)**** |
| 25.0-29.9 | 913 | **0.89 (0.81-0.98)*** | 550 | 0.98 (0.87-1.09) |
| 30.0-34.9 | 1,210 | Reference | 947 | Reference |
| 35.0-39.9 | 359 | 0.93 (0.82-1.05) | 431 | 0.98 (0.87-1.10) |
| ≥40.0 | 39 | **0.59 (0.40-0.87)**** | 184 | 1.00 (0.87-1.16) |
| Values represent the fecundability per categories of age at pursuing pregnancy (95% confidence interval (CI)), as compared to the reference category. Fecundability represents the per-probability of conceiving. Models were analysed using Cox proportional hazards models. Fecundability ratios were derived from the Hazard Ratios of the Cox proportional hazards models. Models were adjusted for participants body mass index, ethnicity, education level, smoking and alcohol consumption. Parity was included in the women’s models.  * p < 0.05, ** p < 0.01, *** p < 0.001 | | | | |

## Table S10. Associations of Age among Women and Men with Fecundability Ratios, Adjusted Model excluding Top 5% of Time to Pregnancy.

|  | **Women** | | **Men** | |
| --- | --- | --- | --- | --- |
|  | N | FR (95% CI) | N | FR (95% CI) |
| Age at pursuing pregnancy, in years | 2,631 |  | 2,122 |  |
| <25.0 | 210 | **0.77 (0.66-0.91)**** | 103 | 0.94 (0.78-1.14) |
| 25.0-29.9 | 866 | 0.93 (0.85-1.02) | 518 | 1.08 (0.97-1.20) |
| 30.0-34.9 | 1,171 | Reference | 911 | Reference |
| 35.0-39.9 | 346 | 0.91 (0.80-1.03) | 413 | 1.00 (0.89-1.13) |
| ≥40.0 | 38 | **0.55 (0.38-0.80)**** | 177 | 0.97 (0.83-1.13) |
| Values represent the fecundability per categories of age at pursuing pregnancy (95% confidence interval (CI)), as compared to the reference category. Fecundability represents the per-probability of conceiving. Models were analysed using Cox proportional hazards models. Fecundability ratios were derived from the Hazard Ratios of the Cox proportional hazards models. Models were adjusted for participants body mass index, ethnicity, education level, smoking and alcohol consumption. Parity was included in the women’s models. The top 5% of time to pregnancy observations were excluded.  * p < 0.05, ** p < 0.01, *** p < 0.001 | | | | |

## Table S11. Associations of Age among Women and Men with Fecundability Ratios, Adjusted Model including only First Episodes.

|  | **Women** | | **Men** | |
| --- | --- | --- | --- | --- |
|  | N | FR (95% CI) | N | FR (95% CI) |
| Age at pursuing pregnancy, in years | 2,554 |  | 2,032 |  |
| <25.0 | 232 | **0.64 (0.54-0.75)***** | 112 | **0.72 (0.58-0.88)**** |
| 25.0-29.9 | 858 | 0.92 (0.84-1.01) | 508 | 1.00 (0.89-1.12) |
| 30.0-34.9 | 1,106 | Reference | 866 | Reference |
| 35.0-39.9 | 324 | 0.94 (0.83-1.07) | 388 | 0.96 (0.85-1.07) |
| ≥40.0 | 34 | **0.56 (0.37-0.85)**** | 158 | 0.99 (0.85-1.16) |
| Values represent the fecundability per categories of age at pursuing pregnancy (95% confidence interval (CI)), as compared to the reference category. Fecundability represents the per-probability of conceiving. Models were analysed using Cox proportional hazards models. Fecundability ratios were derived from the Hazard Ratios of the Cox proportional hazards models. Models were adjusted for participants body mass index, ethnicity, education level, smoking and alcohol consumption. Parity was included in the women’s model. Only the first episodes were included in the model.  * p < 0.05, ** p < 0.01, *** p < 0.001 | | | | |

## Table S12. Associations of Age among Women and Men with Fecundability Ratios, Adjusted Model including Age of Partner.

|  | **Women** | | **Men** | |
| --- | --- | --- | --- | --- |
|  | N | FR (95% CI) | N | FR (95% CI) |
| Age at pursuing pregnancy, in years | 2,234 |  | 2,234 |  |
| <25.0 | 171 | **0.60 (0.49-0.72)***** | 122 | 0.82 (0.66-1.02) |
| 25.0-29.9 | 756 | **0.87 (0.78-0.96)**** | 550 | 1.05 (0.94-1.18) |
| 30.0-34.9 | 1,021 | Reference | 947 | Reference |
| 35.0-39.9 | 267 | 1.04 (0.91-1.20) | 431 | 0.93 (0.82-1.05) |
| ≥40.0 | 19 | 1.26 (0.91-1.73) | 184 | 0.91 (0.78-1.06) |
| Values represent the fecundability per categories of age at pursuing pregnancy (95% confidence interval (CI)), as compared to the reference category. Fecundability represents the per-probability of conceiving. Models were analysed using Cox proportional hazards models. Fecundability ratios were derived from the Hazard Ratios of the Cox proportional hazards models. Models were adjusted for age of partner, body mass index, ethnicity, education level, smoking and alcohol consumption of women and men. Parity was included in the women’s model.  * p < 0.05, ** p < 0.01, *** p < 0.001 | | | | |

**Table S13.** Associations of Age among Women and Men with Fecundability Ratios, Adjusted Model including only Preconceptionally Included Episodes.

|  | **Women** | | **Men** | |
| --- | --- | --- | --- | --- |
|  | N | FR (95% CI) | N | FR (95% CI) |
| Age at pursuing pregnancy, in years | 930 |  | 607 |  |
| <25.0 | 58 | **0.48 (0.33-0.69)***** | 24 | 0.70 (0.44-1.11) |
| 25.0-29.9 | 292 | 0.85 (0.71-1.02) | 128 | 1.01 (0.81-1.26) |
| 30.0-34.9 | 430 | Reference | 296 | Reference |
| 35.0-39.9 | 131 | 0.80 (0.64-1.00) | 104 | 0.90 (0.72-1.12) |
| ≥40.0 | 19 | **0.32 (0.15-68)**** | 55 | 1.07 (0.81-1.39) |
| Values represent the fecundability per categories of age at pursuing pregnancy (95% confidence interval (CI)), as compared to the reference category. Fecundability represents the per-probability of conceiving. Models were analysed using Cox proportional hazards models. Fecundability ratios were derived from the Hazard Ratios of the Cox proportional hazards models. Models were adjusted for participants body mass index, ethnicity, education level, smoking and alcohol consumption. Parity was included in the women’s models. Only preconceptionally included episodes were included in the model.  * p < 0.05, ** p < 0.01, *** p < 0.001 | | | | |

## Table S14. Associations of Joint Age of Women and Men with Fecundability Ratios, Adjusted Model.

|  | **Women and men combined** | |
| --- | --- | --- |
|  | Effect Estimate | p-value |
| Interaction term age women * age men | 0.998 (0.996-0.999) | 0.006 |
|  | N | FR (95% CI) |
| Both aged <35 years at pursuing pregnancy | 1,535 | Reference |
| Only partner aged ≥35 years at pursuing pregnancy | 413 | 1.05 (0.94-1.17) |
| Only woman aged ≥35 years at pursuing pregnancy | 84 | 1.23 (0.97-1.56) |
| Both aged ≥35 years at pursuing pregnancy | 202 | 1.08 (0.94-1.25) |
| Values represent the fecundability per categories of age at pursuing pregnancy (95% confidence interval (CI)), as compared to the reference category Fecundability represents the per-probability of conceiving. Models were analysed using Cox proportional hazards models. Fecundability ratios were derived from the Hazard Ratios of the Cox proportional hazards models. Models were adjusted for body mass index, ethnicity, education level, smoking and alcohol consumption of women and men, and parity.  * p < 0.05, ** p < 0.01, *** p < 0.001 | | |

# Supplemental Tables of Associations of Age among Women and Men with Odds of Infertility.

## Table S15. Associations of Age among Women and Men with Odds of Infertility, Basic Model.

|  | **Women** | | **Men** | |
| --- | --- | --- | --- | --- |
|  | N | OR (95% CI) | N | OR (95% CI) |
| Age at pursuing pregnancy, in years | 3,032 |  | 2,462 |  |
| <25.0 | 276 | **2.50 (1.91-3.26)***** | 134 | **1.73 (1.18-2.52)**** |
| 25.0-29.9 | 995 | **1.28 (1.07-1.52)**** | 605 | 1.03 (0.82-1.30) |
| 30.0-34.9 | 1,322 | Reference | 1,033 | Reference |
| 35.0-39.9 | 395 | **1.30 (1.02-1.65)*** | 479 | 1.04 (0.80-1.34) |
| ≥40.0 | 44 | **2.51 (1.35-4.65)**** | 211 | 1.14 (0.81-1.62) |
| Values represent the odds of infertility (time to pregnancy > 12 months) per categories of age at pursuing pregnancy (95% confidence interval (CI)) from logistic regression models, as compared to the reference category.  * p < 0.05, ** p < 0.01, *** p < 0.001 | | | | |

## Table S16. Associations of Age among Women and Men with Odds of Infertility, Adjusted Model.

|  | **Women** | | **Men** | |
| --- | --- | --- | --- | --- |
|  | N | OR (95% CI) | N | OR (95% CI) |
| Age at pursuing pregnancy, in years | 3,032 |  | 2,462 |  |
| <25.0 | 276 | **2.14 (1.60-2.87)***** | 134 | 1.46 (0.99-2.17) |
| 25.0-29.9 | 995 | **1.27 (1.05-1.53)*** | 605 | 0.97 (0.77-1.23) |
| 30.0-34.9 | 1,322 | Reference | 1033 | Reference |
| 35.0-39.9 | 395 | 1.10 (0.86-1.40) | 479 | 1.02 (0.79-1.32) |
| ≥40.0 | 44 | **2.00 (1.05-3.80)*** | 211 | 1.05 (0.74-1.49) |
| Values represent the odds of infertility (time to pregnancy > 12 months) per categories of age at pursuing pregnancy (95% confidence interval (CI)) from logistic regression models, as compared to the reference category. Models were adjusted for body mass index, ethnicity, education level, smoking and alcohol consumption. Parity was included in the women’s model.  * p < 0.05, ** p < 0.01, *** p < 0.001 | | | | |

## Table S17. Associations of Age among Women and Men with Odds of Infertility, Adjusted Model excluding Couples undergoing Assisted Reproductive Technology.

|  | **Women** | | **Men** | |
| --- | --- | --- | --- | --- |
|  | N | OR (95% CI) | N | OR (95% CI) |
| Age at pursuing pregnancy, in years | 2,735 |  | 2,232 |  |
| <25.0 | 248 | **2.45 (1.78-3.37)***** | 122 | 1.54 (0.99-2.38) |
| 25.0-29.9 | 905 | **1.42 (1.15-1.75)***** | 550 | 0.91 (0.69-1.20) |
| 30.0-34.9 | 1,193 | Reference | 945 | Reference |
| 35.0-39.9 | 350 | 1.06 (0.79-1.40) | 431 | 0.97 (0.71-1.32) |
| ≥40.0 | 39 | **2.12 (1.07-4.20)*** | 184 | 0.84 (0.56-1.26) |
| Values represent the odds of infertility (time to pregnancy > 12 months) per categories of age at pursuing pregnancy (95% confidence interval (CI)) from logistic regression models, as compared to the reference category. Models were adjusted for age, ethnicity, education level, smoking, alcohol consumption, and parity in the women’s model, and stratified for assisted reproductive technology.  * p < 0.05, ** p < 0.01, *** p < 0.001 | | | | |

## Table S18. Associations of Age among Women and Men with Odds of Infertility, Adjusted Model excluding Top 5% of Time to Pregnancy.

|  | **Women** | | **Men** | |
| --- | --- | --- | --- | --- |
|  | N | OR (95% CI) | N | OR (95% CI) |
| Age at pursuing pregnancy, in years | 2,880 |  | 2,338 |  |
| <25.0 | 237 | **1.73 (1.26-2.37)**** | 114 | 1.10 (0.70-1.72) |
| 25.0-29.9 | 937 | 1.17 (0.97-1.42) | 577 | 0.97 (0.75-1.25) |
| 30.0-34.9 | 1,277 | Reference | 986 | Reference |
| 35.0-39.9 | 386 | 1.20 (0.94-1.54) | 460 | 1.08 (0.82-1.41) |
| ≥40.0 | 43 | **2.21 (1.16-4.19)*** | 201 | 1.08 (0.75-1.56) |
| Values represent the odds of infertility (time to pregnancy > 12 months) per categories of age at pursuing pregnancy (95% confidence interval (CI)) from logistic regression models, as compared to the reference category. Models were adjusted for age, ethnicity, education level, smoking and alcohol consumption. Parity was included in the women’s model. The top 5% of time to pregnancy observations were excluded.  * p < 0.05, ** p < 0.01, *** p < 0.001 | | | | |

## Table S19. Associations of Age among Women and Men with Odds of Infertility, Adjusted Model including only First Episodes.

|  | **Women** | | **Men** | |
| --- | --- | --- | --- | --- |
|  | N | OR (95% CI) | N | OR (95% CI) |
| Age at pursuing pregnancy, in years | 2,793 |  | 2,237 |  |
| <25.0 | 256 | **2.09 (1.55-2.83)***** | 123 | 1.49 (0.98-2.24) |
| 25.0-29.9 | 934 | **1.23 (1.01-1.49)*** | 557 | 0.93 (0.72-1.20) |
| 30.0-34.9 | 1,205 | Reference | 943 | Reference |
| 35.0-39.9 | 359 | 1.11 (0.86-1.44) | 433 | 1.13 (0.87-1.47) |
| ≥40.0 | 39 | **2.47 (1.27-4.83)**** | 181 | 1.10 (0.77-1.58) |
| Values represent the odds of infertility (time to pregnancy > 12 months) per categories of age at pursuing pregnancy (95% confidence interval (CI)) from logistic regression models, as compared to the reference category. Models were adjusted for age, ethnicity, education level, smoking and alcohol consumption. Parity was included in the women’s model. Only the first episodes were included in the model.  * p < 0.05, ** p < 0.01, *** p < 0.001 | | | | |

## Table S20. Associations of Age among Women and Men with Odds of Infertility, Adjusted Model including Age of Partner.

|  | **Women** | | **Men** | |
| --- | --- | --- | --- | --- |
|  | N | OR (95% CI) | N | OR (95% CI) |
| Age at pursuing pregnancy, in years | 2,462 |  | 2,462 |  |
| <25.0 | 195 | **3.00 (2.05-4.39)***** | 134 | 1.01 (0.65-1.57) |
| 25.0-29.9 | 828 | **1.47 (1.18-1.84)***** | 605 | 0.82 (0.64-1.05) |
| 30.0-34.9 | 1,123 | Reference | 1,033 | Reference |
| 35.0-39.9 | 294 | 0.87 (0.63-1.20) | 479 | 1.17 (0.90-1.53) |
| ≥40.0 | 22 | 0.57 (0.19-1.71) | 211 | 1.32 (0.91-1.91) |
| Values represent the odds of infertility (time to pregnancy > 12 months) per categories of age at pursuing pregnancy (95% confidence interval (CI)) from logistic regression models, as compared to the reference category. Models were adjusted for age of partner, age, ethnicity, education level, smoking and alcohol consumption. Parity was included in the women’s model.  * p < 0.05, ** p < 0.01, *** p < 0.001 | | | | |

**Table S21.** Associations of Age among Women and Men with Odds of Infertility, Adjusted Model including only Preconceptionally Included Episodes.

|  | **Women** | | **Men** | |
| --- | --- | --- | --- | --- |
|  | N | OR (95% CI) | N | OR (95% CI) |
| Age at pursuing pregnancy, in years | 1,026 |  | 707 |  |
| <25.0 | 67 | **3.00 (1.55-5.82)***** | 29 | 1.12 (0.50-2.48) |
| 25.0-29.9 | 325 | 1.34 (0.99-1.81) | 151 | 0.98 (0.64-1.50) |
| 30.0-34.9 | 474 | Reference | 336 | Reference |
| 35.0-39.9 | 140 | 1.12 (0.76-1.65) | 123 | 1.06 (0.68-1.66) |
| ≥40.0 | 20 | **4.03 (1.29-12.59)*** | 68 | 1.25 (0.71-2.19) |
| Values represent the odds of infertility (time to pregnancy > 12 months) per categories of age at pursuing pregnancy (95% confidence interval (CI)) from logistic regression models, as compared to the reference category. Models were adjusted for age, ethnicity, education level, smoking and alcohol consumption. Parity was included in the women’s model. Only preconceptionally included episodes were included in the model.  * p < 0.05, ** p < 0.01, *** p < 0.001 | | | | |

## Table S22. Associations of Joint Age of Women and Men with Odds of Infertility, Adjusted Model.

| **Total population** |  | |
| --- | --- | --- |
|  | **Men** | |
| **Women** | <35 years | ≥35 years |
| <35 years | 1,679 (457) | 469 (136) |
| ≥35 years | 95 (27) | 221 (54) |
| * NA = 1,572 of 4,036 episodes | | |
| **Time to pregnancy population , stratified for infertility (>12 months)** | | |
|  | **Men** | |
| **Women** | <35 years | ≥35 years |
| <35 years | 1,677 (457) | 469 (136) |
| ≥35 years | 95 (27) | 221 (54) |
| * NA = 5,70 of 3,032 episodes | | |

|  | **Women and men combined** | |
| --- | --- | --- |
|  | OR (95% CI) | p-value |
| Interaction term age women * age men | 1.00 (0.999-1.005) | 0.19 |
|  | N | OR (95% CI) |
| Both aged <35 years at pursuing pregnancy | 1,677 | Reference |
| Only partner aged ≥35 years at pursuing pregnancy | 469 | 1.08 (0.84-1.38) |
| Only woman aged ≥35 years at pursuing pregnancy | 95 | 0.95 (0.59-1.54) |
| Both aged ≥35 at pursuing pregnancy | 221 | 0.73 (0.52-1.04) |
| Values represent the odds of infertility per categories of age at pursuing pregnancy (95% confidence interval (CI)) from logistic regression models, as compared to the reference category. Models were adjusted for age, ethnicity, education level, smoking and alcohol consumption of both women and men, and parity.  * p < 0.05, ** p < 0.01, *** p < 0.001 | | |

# Supplemental Tables of Associations of Age among Women and Men with Hazard Ratios of Miscarriage.

## Table S23. Associations of Age among Women and Men with Hazard Ratios of Miscarriage, Basic Model.

|  | **Women** | | **Men** | |
| --- | --- | --- | --- | --- |
|  | N | HR (95% CI) | N | HR (95% CI) |
| Age at conception, in years | 2,824 |  | 2,387 |  |
| <25.0 | 185 | **0.55 (0.30-1.00)*** | 97 | 0.96 (0.50-1.86) |
| 25.0-29.9 | 768 | 1.01 (0.76-1.34) | 482 | 0.90 (0.62-1.30) |
| 30.0-34.9 | 1,287 | Reference | 947 | Reference |
| 35.0-39.9 | 507 | **1.92 (1.48-2.50)***** | 577 | 1.07 (0.77-1.50) |
| ≥40.0 | 77 | **3.81 (2.41-6.03)***** | 284 | **1.89 (1.30-2.76)***** |
| Values represent the Hazard Ratio of miscarriage per categories of age at conception (95% confidence interval (CI)), as compared to the reference category. Models were analysed using Cox proportional hazards models.  * p < 0.05, ** p < 0.01, *** p < 0.001 | | | | |

## Table S24. Associations of Age among Women and Men with Hazard Ratios of Miscarriage, Adjusted Model.

|  | **Women** | | **Men** | |
| --- | --- | --- | --- | --- |
|  | N | HR (95% CI) | N | HR (95% CI) |
| Age at conception, in years | 2,824 |  | 2,387 |  |
| <25.0 | 185 | 0.56 (0.30-1.03) | 97 | 0.99 (0.51-1.93) |
| 25.0-29.9 | 768 | 1.01 (0.77-1.34) | 482 | 0.89 (0.62-1.29) |
| 30.0-34.9 | 1,287 | Reference | 947 | Reference |
| 35.0-39.9 | 507 | **1.91 (1.46-2.50)***** | 577 | 1.11 (0.79-1.56) |
| ≥40.0 | 77 | **3.60 (2.30-5.61)***** | 284 | **1.92 (1.31-2.81)***** |
| Values represent the Hazard Ratio of miscarriage per categories of age at conception (95% confidence interval (CI)) as compared to the reference category. Models were analysed using Cox proportional hazards models. Models were adjusted for body mass index, ethnicity, education level, smoking and alcohol consumption. Parity and history of miscarriage were included in the women’s model.  * p < 0.05, ** p < 0.01, *** p < 0.001 | | | | |

## Table S25. Associations of Age among Women and Men with Hazard Ratios of Miscarriage, Adjusted Model excluding Couples undergoing Assisted Reproductive Technology.

|  | **Women** | | **Men** | |
| --- | --- | --- | --- | --- |
|  | N | HR (95% CI) | N | HR (95% CI) |
| Age at conception, in years | 2,559 |  | 2,183 |  |
| <25.0 | 182 | **0.52 (0.28-0.97)*** | 96 | 0.95 (0.49-1.85) |
| 25.0-29.9 | 721 | 0.98 (0.74-1.31) | 457 | 0.87 (0.60-1.27) |
| 30.0-34.9 | 1,173 | Reference | 887 | Reference |
| 35.0-39.9 | 425 | **1.75 (1.31-2.34)***** | 508 | 1.10 (0.77-1.56) |
| ≥40.0 | 58 | **4.47 (2.85-7.01)***** | 235 | **1.96 (1.30-2.94)**** |
| Values represent the Hazard Ratio of miscarriage per categories of age at conception (95% confidence interval (CI)) as compared to the reference category. Models were analysed using Cox proportional hazards models. Models were adjusted for age, ethnicity, education level, smoking, alcohol consumption, parity and history of miscarriage in the women’s model, and stratified for assisted reproductive technology.  * p < 0.05, ** p < 0.01, *** p < 0.001 | | | | |

## Table S26. Associations of Age among Women and Men with Hazard Ratios of Miscarriage, Adjusted Model including only First Episodes.

|  | **Women** | | **Men** | |
| --- | --- | --- | --- | --- |
|  | N | HR (95% CI) | N | HR (95% CI) |
| Age at conception, in years | 2,509 |  | 2,117 |  |
| <25.0 | 176 | 0.55 (0.28-1.08) | 92 | 1.14 (0.57-2.28) |
| 25.0-29.9 | 717 | 1.06 (0.79-1.43) | 447 | 0.95 (0.64-1.41) |
| 30.0-34.9 | 1,129 | Reference | 848 | Reference |
| 35.0-39.9 | 428 | **1.97 (1.46-2.65)***** | 497 | 1.08 (0.74-1.58) |
| ≥40.0 | 59 | **3.34 (2.03-5.49)***** | 233 | **1.88 (1.26-2.80)**** |
| Values represent the Hazard Ratio of miscarriage per categories of age at conception (95% confidence interval (CI)) as compared to the reference category. Models were analysed using Cox proportional hazards models. Models were adjusted for age, ethnicity, education level, smoking and alcohol consumption. Parity and history of miscarriage were included in the women’s model. Only the first episodes were included in the model.  * p < 0.05, ** p < 0.01, *** p < 0.001 | | | | |

## Table S27. Associations of Age among Women and Men with Hazard Ratios of Miscarriage, Adjusted Model including Age of Partner.

|  | **Women** | | **Men** | |
| --- | --- | --- | --- | --- |
|  | N | HR (95% CI) | N | HR (95% CI) |
| Age at conception, in years | 2,387 |  | 2,387 |  |
| <25.0 | 138 | 0.64 (0.29-1.43) | 92 | 1.43 (0.71-2.87) |
| 25.0-29.9 | 669 | 1.22 (0.89-1.69) | 447 | 1.03 (0.70-1.51) |
| 30.0-34.9 | 1,134 | Reference | 848 | Reference |
| 35.0-39.9 | 400 | **1.68 (1.19-2.37)**** | 497 | 0.97 (0.70-1.36) |
| ≥40.0 | 46 | **3.33 (1.70-6.54)***** | 233 | **1.49 (1.00-2.22)*** |
| Values represent the Hazard Ratio of miscarriage per categories of age at conception (95% confidence interval (CI)) as compared to the reference category. Models were analysed using Cox proportional hazards models. Models were adjusted for age of partner, age, ethnicity, education level, smoking and alcohol consumption. Parity and history of miscarriage were included in the women’s model.  * p < 0.05, ** p < 0.01, *** p < 0.001 | | | | |

**Table S28.** Associations of Age among Women and Men with Hazard Ratios of Miscarriage, Adjusted Model including only Preconceptionally Included Episodes.

|  | **Women** | | **Men** | |
| --- | --- | --- | --- | --- |
|  | N | HR (95% CI) | N | HR (95% CI) |
| Age at conception, in years | 823 |  | 691 |  |
| <25.0 | 15 | 0.46 (0.06-3.74) | 11 | 1.46 (0.35-6.08) |
| 25.0-29.9 | 192 | 1.06 (0.68-1.64) | 127 | 0.67 (0.36-1.26) |
| 30.0-34.9 | 421 | Reference | 303 | Reference |
| 35.0-39.9 | 174 | **1.66 (1.12-2.47)*** | 165 | 1.14 (0.68-1.90) |
| ≥40.0 | 21 | **4.15 (2.18-7.88)***** | 85 | **1.77 (1.02-3.08)*** |
| Values represent the Hazard Ratio of miscarriage per categories of age at conception (95% confidence interval (CI)) as compared to the reference category. Models were analysed using Cox proportional hazards models. Models were adjusted for age, ethnicity, education level, smoking and alcohol consumption. Parity and history of miscarriage were included in the women’s model. Only preconceptionally included episodes were included in the model.  * p < 0.05, ** p < 0.01, *** p < 0.001 | | | | |

## Table S29. Associations of Joint Age of Women and Men with Hazard Ratios of Miscarriage, Adjusted Model.

|  | **Women and men combined** | |
| --- | --- | --- |
|  | Effect Estimate | p-value |
| Interaction term age women * age men | 1.00 (1.00-1.01) | 0.008 |
|  | N | HR (95% CI) |
| Both aged <35years at conception | 1,422 | Reference |
| Only partner aged ≥35 years at conception | 519 | 1.03 (0.73-1.44) |
| Only woman aged ≥35 years at conception | 104 | 1.36 (0.76-2.43) |
| Both aged ≥35 years at conception | 342 | **2.18 (1.53-3.10)***** |
| Values represent the Hazard Ratio of miscarriage per categories of age at conception (95% confidence interval (CI)) as compared to the reference category. Models were analysed using Cox proportional hazards models. Models were adjusted for body mass index, ethnicity, education level, smoking and alcohol consumption of both women and men, and parity and history of miscarriage.  * p < 0.05, ** p < 0.01, *** p < 0.001 | | |

# Supplemental Tables of Associations of Age among Women and Men with Odds of Miscarriage.

## Table S30. Associations of Age among Women and Men with Odds of Miscarriage, Basic Model.

|  | **Women** | | **Men** | |
| --- | --- | --- | --- | --- |
|  | N | OR (95% CI) | N | OR (95% CI) |
| Age at conception, in years | 2,831 |  | 2,392 |  |
| <25.0 | 186 | 0.58 (0.32-1.05) | 98 | 1.10 (0.56-2.13) |
| 25.0-29.9 | 771 | 1.03 (0.77-1.38) | 483 | 0.92 (0.63-1.35) |
| 30.0-34.9 | 1,289 | Reference | 947 | Reference |
| 35.0-39.9 | 508 | **2.04 (1.53-2.70)***** | 579 | 1.09 (0.77-1.55) |
| ≥40.0 | 77 | **4.54 (2.60-7.94)***** | 285 | **2.05 (1.36-3.08)***** |
| Values represent the odds of miscarriage per categories of age at conception (95% confidence interval (CI)) from logistic regression models, as compared to the reference category.  * p < 0.05, ** p < 0.01, *** p < 0.001 | | | | |

## Table S31. Associations of Age among Women and Men with Odds of Miscarriage, Adjusted Model.

|  | **Women** | | **Men** | |
| --- | --- | --- | --- | --- |
|  | N | OR (95% CI) | N | OR (95% CI) |
| Age at conception, in years | 2,831 |  | 2,392 |  |
| <25.0 | 186 | 0.56 (0.30-1.04) | 98 | 1.10 (0.56-2.17) |
| 25.0-29.9 | 771 | 1.02 (0.76-1.38) | 483 | 0.91 (0.62-1.34) |
| 30.0-34.9 | 1,289 | Reference | 947 | Reference |
| 35.0-39.9 | 508 | **2.03 (1.51-2.72)***** | 579 | 1.13 (0.79-1.61) |
| ≥40.0 | 77 | **4.24 (2.45-7.36)***** | 285 | **2.09 (1.39-3.14)***** |
| Values represent the odds of miscarriage per categories of age at conception (95% confidence interval (CI)) from logistic regression models, as compared to the reference category. Models were adjusted for body mass index, ethnicity, education level, smoking and alcohol consumption. Parity and history of miscarriage were included in the women’s model.  * p < 0.05, ** p < 0.01, *** p < 0.001 | | | | |

## Table S32. Associations of Age among Women and Men with Odds of Miscarriage, Adjusted Model excluding Couples undergoing Assisted Reproductive Technology.

|  | **Women** | | **Men** | |
| --- | --- | --- | --- | --- |
|  | N | OR (95% CI) | N | OR (95% CI) |
| Age at conception, in years | 2,566 |  | 2,188 |  |
| <25.0 | 183 | **0.53 (0.28-0.98)*** | 97 | 1.30 (0.63-2.65) |
| 25.0-29.9 | 724 | 0.99 (0.73-1.33) | 458 | 0.96 (0.63-1.46) |
| 30.0-34.9 | 1,175 | Reference | 887 | Reference |
| 35.0-39.9 | 426 | **1.85 (1.35-2.53)***** | 510 | 1.11 (0.75-1.64) |
| ≥40.0 | 58 | **5.83 (3.19-10.62)***** | 236 | **2.08 (1.35-3.20)***** |
| Values represent the odds of miscarriage per categories of age at conception (95% confidence interval (CI)) from logistic regression models, as compared to the reference category. Models were adjusted for body mass index, ethnicity, education level, smoking, alcohol consumption, parity and history of miscarriage in the women’s model, and stratified for assisted reproductive technology.  * p < 0.05, ** p < 0.01, *** p < 0.001 | | | | |

## Table S33. Associations of Age among Women and Men with Odds of Miscarriage, Adjusted Model including only First Episodes.

|  | **Women** | | **Men** | |
| --- | --- | --- | --- | --- |
|  | N | OR (95% CI) | N | OR (95% CI) |
| Age at conception, in years | 2,515 |  | 2,121 |  |
| <25.0 | 176 | 0.52 (0.26-1.03) | 93 | 1.30 (0.63-2.65) |
| 25.0-29.9 | 720 | 1.08 (0.79-1.47) | 447 | 0.96 (0.63-1.46) |
| 30.0-34.9 | 1,131 | Reference | 848 | Reference |
| 35.0-39.9 | 429 | **2.08 (1.51-2.87)***** | 499 | 1.11 (0.75-1.64) |
| ≥40.0 | 59 | **3.74 (2.07-6.73)***** | 234 | **2.08 (1.35-3.20)***** |
| Values represent the odds of miscarriage per categories of age at conception (95% confidence interval (CI)) from logistic regression models, as compared to the reference category. Models were adjusted for age, ethnicity, education level, smoking and alcohol consumption. Parity and history of miscarriage were included in the women’s model. Only the first episodes were included in the model.  * p < 0.05, ** p < 0.01, *** p < 0.001 | | | | |

## Table S34. Associations of Age among Women and Men with Odds of Miscarriage, Adjusted Model including Age of Partner.

|  | **Women** | | **Men** | |
| --- | --- | --- | --- | --- |
|  | N | OR (95% CI) | N | OR (95% CI) |
| Age at conception, in years | 2,392 |  | 2,392 |  |
| <25.0 | 139 | 0.67 (0.31-1.45) | 98 | 1.61 (0.79-3.31) |
| 25.0-29.9 | 671 | 1.23 (0.87-1.72) | 483 | 1.05 (0.71-1.57) |
| 30.0-34.9 | 1,135 | Reference | 947 | Reference |
| 35.0-39.9 | 401 | **1.77 (1.23-2.54)**** | 579 | 0.98 (0.69-1.39) |
| ≥40.0 | 46 | **3.83 (1.74-8.42)***** | 285 | **1.61 (1.05-2.47)*** |
| Values represent the odds of miscarriage per categories of age at conception (95% confidence interval (CI)) from logistic regression models, as compared to the reference category. Models were adjusted for age of partner, body mass index, ethnicity, education level, smoking and alcohol consumption. Parity and history of miscarriage were included in the women’s model.  * p < 0.05, ** p < 0.01, *** p < 0.001 | | | | |

**Table S35.** Associations of Age among Women and Men with Odds of Miscarriage, Adjusted Model including only Preconceptionally Included Episodes.

|  | **Women** | | **Men** | |
| --- | --- | --- | --- | --- |
|  | N | OR (95% CI) | N | OR (95% CI) |
| Age at conception, in years | 826 |  | 692 |  |
| <25.0 | 15 | 0.40 (0.05-3.50) | 11 | 1.55 (0.32-7.58) |
| 25.0-29.9 | 194 | 1.12 (0.69-1.81) | 127 | 0.66 (0.34-1.28) |
| 30.0-34.9 | 422 | Reference | 303 | Reference |
| 35.0-39.9 | 174 | **1.78 (1.14-2.77)*** | 165 | 1.16 (0.67-2.01) |
| ≥40.0 | 21 | **5.66 (2.35-13.62)***** | 86 | **2.04 (1.11-3.75)*** |
| Values represent the odds of miscarriage per categories of age at conception (95% confidence interval (CI)) from logistic regression models, as compared to the reference category. Models were adjusted for age, ethnicity, education level, smoking and alcohol consumption. Parity and history of miscarriage were included in the women’s model. Only preconceptionally included episodes were included in the model.  * p < 0.05, ** p < 0.01, *** p < 0.001 | | | | |

## Table S36. Associations of Joint Age of Women and Men with Odds of Miscarriage, Adjusted Model.

| **Total population** |  | |
| --- | --- | --- |
|  | **Men** | |
| **Women** | <35 years | ≥35 years |
| <35 years | 1,740 (129) | 614 (47) |
| ≥35 years | 126 (12) | 423 (62) |
| * NA = 1,133 of 4,036 episodes | | |
| **Miscarriage population, stratified for miscarriage** |  | |
|  | **Men** | |
| **Women** | <35 years | ≥35 years |
| <35 years | 1,424 (129) | 521 (47) |
| ≥35 years | 104 (12) | 343 (62) |
| * NA = 439 of 2,770 episodes | | |

|  | **Women and men combined** | |
| --- | --- | --- |
|  | Effect Estimate | p-value |
| Interaction term age women * age men | 1.01 (1.00-1.01) | 0.004 |
|  | N | OR (95% CI) |
| Both aged <35 years at conception | 1,424 | Reference |
| Only partner aged ≥35 years at conception | 521 | 1.02 (0.72-1.45) |
| Only woman aged ≥35 years at conception | 104 | 1.33 (0.72-2.46) |
| Both aged ≥35 years at conception | 343 | **2.33 (1.61-3.37)***** |
| Values represent the odds of miscarriage per categories of age at conception (95% confidence interval (CI)) from logistic regression models, as compared to the reference category. Models were adjusted for body mass index, ethnicity, education level, smoking and alcohol consumption of both women and men, and parity and history of miscarriage.  * p < 0.05, ** p < 0.01, *** p < 0.001 | | |
